# Emergence of CD4+ and CD8+ Polyfunctional T Cell Responses Against Immunodominant Lytic and Latent EBV Antigens in Children With Primary EBV Infection

**DOI:** 10.3389/fmicb.2018.00416

**Published:** 2018-03-07

**Authors:** Janice K. P. Lam, K. F. Hui, Raymond J. Ning, X. Q. Xu, K. H. Chan, Alan K. S. Chiang

**Affiliations:** ^1^Department of Paediatrics and Adolescent Medicine, Li Ka Shing Faculty of Medicine, Queen Mary Hospital, The University of Hong Kong, Pokfulam, Hong Kong; ^2^Department of Microbiology, Li Ka Shing Faculty of Medicine, Queen Mary Hospital, The University of Hong Kong, Pokfulam, Hong Kong

**Keywords:** EBV, polyfunctional T cells, immunodominance, infectious mononucleosis, asymptomatic primary infection

## Abstract

Long term carriers were shown to generate robust polyfunctional T cell (PFC) responses against lytic and latent antigens of Epstein-Barr virus (EBV). However, the time of emergence of PFC responses against EBV antigens, pattern of immunodominance and difference between CD4+ and CD8+ T cell responses during various stages of EBV infection are not clearly understood. A longitudinal study was performed to assess the development of antigen-specific PFC responses in children diagnosed to have primary symptomatic (infectious mononucleosis [IM]) and asymptomatic (AS) EBV infection. Evaluation of IFN-γ secreting CD8+ T cell responses upon stimulation by HLA class I-specific peptides of EBV lytic and latent proteins by ELISPOT assay followed by assessment of CD4+ and CD8+ PFC responses upon stimulation by a panel of overlapping EBV peptides for co-expression of IFN-γ, TNF-α, IL-2, perforin and CD107a by flow cytometry were performed. Cytotoxicity of T cells against autologous lymphoblastoid cell lines (LCLs) as well as EBV loads in PBMC and plasma were also determined. Both IM and AS patients had elevated PBMC and plasma viral loads which declined steadily during a 12-month period from the time of diagnosis whilst decrease in the magnitude of CD8+ T cell responses toward EBV lytic peptides in contrast to increase toward latent peptides was shown with no significant difference between those of IM and AS patients. Both lytic and latent antigen-specific CD4+ and CD8+ T cells demonstrated polyfunctionality (defined as greater or equal to three functions) concurrent with enhanced cytotoxicity against autologous LCLs and steady decrease in plasma and PBMC viral loads over time. Immunodominant peptides derived from BZLF1, BRLF1, BMLF1 and EBNA3A-C proteins induced the highest proportion of CD8+ as well as CD4+ PFC responses. Diverse functional subtypes of both CD4+ and CD8+ PFCs were shown to emerge at 6–12 months. In conclusion, EBV antigen-specific CD4+ and CD8+ PFC responses emerge during the first year of primary EBV infection, with greatest responses toward immunodominant epitopes in both lytic and latent proteins, correlating to steady decline in PBMC and plasma viral loads.

## Introduction

Epstein-Barr virus (EBV) is a human gamma-1 herpes virus which infects more than 95% of the population (Young and Rickinson, 2004). This virus is strongly associated with a range of lymphoid and epithelial malignancies and autoimmune diseases (Longnecker and Neipel, 2007; Posnett, 2008; Salvetti et al., 2009). EBV preferentially infects naïve B cells at oropharyngeal lymphoid tissues and subsequently establishes a persistent infection in the circulating memory B cells (Babcock et al., 1998; Rickinson et al., 2014). Once EBV establishes a latent infection, it downregulates the expression of most of the viral genes thereby evading the host immune responses (Kurth et al., 2000). Primary EBV infection typically occurs during childhood without apparent clinical symptoms and evolves into a non-symptomatic persistent virus carrier state (Hislop et al., 2007). Delayed primary infection in some individuals leads to the development of a self-limiting disease called infectious mononucleosis (IM) (Krabbe et al., 1981; Sumaya and Ench, 1985; Niedobitek et al., 1997; Rickinson et al., 2014). EBV has transforming capability as manifested by the occurrence of uncontrolled malignant B cell proliferation in immunosuppressed individuals such as solid organ or stem cell transplantation recipients (Murukesan and Mukherjee, 2012).

The understanding of the mechanisms by which the human immune system controls EBV infection is incomplete due to the difficulties in identifying specific EBV peptides across a wide range of HLA alleles and the fact that EBV can express a large number of lytic and latent proteins displaying hierarchy of immunodominance in its life cycle (Pudney et al., 2005). IM, which is characterized by large expansions of CD8+ T cells and NK cells, provides an excellent model for the study of immune responses against EBV (Callan et al., 1996; Rickinson et al., 2014). The current knowledge on EBV-specific T-cell immunity is mostly derived from studies of IM peripheral blood mononuclear cell (PBMC) samples using either MHC-specific tetramers to determine the percentages of EBV-specific T cells or Enzyme-linked immunosorbent spot (ELISPOT) assay to detect the production of interferon-gamma (IFN-γ) by EBV-specific CD8+ T cells (McCutcheon et al., 1997; Schmittel et al., 1997; Helms et al., 2000; Jennes et al., 2002). However, the functional roles of CD4+ and CD8+ T cells in the control of EBV infection are not completely understood.

Polyfunctional T cells (PFC) are T cells which have multiple functions, such as degranulation of cytotoxic proteins and production of multiple cytokines such as IFN-γ, tumor necrosis factor-alpha (TNF-α), interleukin-2 (IL-2) simultaneously. Accumulating evidence suggests that PFC are associated with more effective control of chronic microbial infections including human immunodeficiency virus (HIV), hepatitis C virus (HCV), and cytomegalovirus (CMV) (Harari et al., 2005; Casazza et al., 2006; Ciuffreda et al., 2008; Duvall et al., 2008; Qiu et al., 2012). We and other groups have studied EBV-specific PFC in long term carriers and found that they produced more cytokines per cell than the single functional T cells and may be functionally superior (Smith et al., 2009; Ning et al., 2011). Another study found that frequencies of PFC were higher in HIV non-progressors than in progressors (Betts et al., 2006). Interestingly, a recent study demonstrated that polyfunctional and IFN-γ mono-functional CD4+ T cells are molecularly distinct and the polyfunctional gene signatures in response to *Plasmodium falciparum* infection and influenza infection were highly conserved (Burel et al., 2017). These findings support the notion that PFC contribute to more robust T-cell immunity in the control of virus infections. However, how PFC arise during primary EBV infection and evolve over time as well as their role in the long term control of EBV from primary infection stage to long term persistence remain unclear.

Here, we conducted a longitudinal study to assess the development and maturation of T cell responses to EBV from acute infection stage to long term persistence in two primary infection cohorts in children, those presenting as IM and those as asymptomatic primary infection (AS). ELISPOT assay was first performed to detect the IFN-γ secreting CD8+ T cell responses upon stimulation by HLA class I-specific peptides of EBV lytic and latent proteins in 18 longitudinally followed IM cases and 12 AS cases. A 9-color flow cytometric assay which simultaneously delineates five parameters: production of IFN-γ, perforin, TNF-α and IL-2, and surface mobilization of CD107a (degranulation marker), upon stimulation by overlapping peptide pools of 4 EBV lytic and 5 latent cycle proteins was then performed to further evaluate the EBV-specific CD4+ and CD8+ PFC responses in another 11 IM cases. Corresponding PBMC and plasma viral loads were determined as measurement of viral control. T cell lysis against autologous lymphoblastoid cell line (LCL) in three IM patients was measured to assess the cytotoxic function of the EBV-specific T cells.

## Materials and Methods

### Subject Recruitment

Two cohorts of study subjects consisting of 29 children with infectious mononucleosis (IM) and 12 with asymptomatic primary infection (AS) were recruited. Serological screening for EBV was performed to confirm their primary infection state of EBV (Supplementary Table 1). Children with positive viral capsid antigen (VCA)-IgM, VCA-IgG, negative EBNA1 IgG and showed clinical symptoms were identified as IM subjects. For those who showed a serological profile of primary EBV infection with positive or negative VCA-IgM, positive VCA-IgG, negative EBNA1 and low VCA-IgG avidity without symptoms were recruited as AS subjects. As the maturation of VCA-IgG antibody from low to high avidity takes up to 6 months, AS patients were estimated to have been infected by EBV within a period of 6 months. Heparinized peripheral blood samples were collected at the time of first examination and subsequently, at 1, 2–5, and 6–12 months after diagnosis for the longitudinal study. Plasma was isolated and stored in -80°C until use. Peripheral blood mononuclear cells (PBMC) were isolated by standard Ficoll-Hypaque density gradient method. Collected PBMC were cryopreserved in fetal bovine serum (FBS) (Invitrogen, Carlsbad, CA, United States) with 10% DMSO under liquid nitrogen until use. All patient samples were handled as potential biohazardous material following the institutional safety procedures. The study protocol was approved by the Institutional Review Board of the University of Hong Kong. Informed written consent was obtained from each participant prior to the study.

#### DNA Extraction, HLA Typing and Quantitative PCR (qPCR)

Qiagen DNeasy blood mini kit (Qiagen) and NucliSENS easyMag instrument (BioMerieux) were used to extract DNA from PBMC and plasma, respectively, in accordance to the manufacturer’s instruction. Part of the DNA samples were transferred to The University of Birmingham and the HLA laboratory in Queen Mary Hospital, The University of Hong Kong for HLA typing. The HLA types of all subjects recruited in ELISPOT assay were documented (Supplementary Table 3). The rest of the DNA was used to measure the EBV loads by qPCR with ABI PRISM 7900 sequence detector (Applied Biosystems, United States). EBV loads in PBMC were determined by the amplification of viral DNA polymerase (Pol, BALF5) sequence. Human β2 microglobulin sequence was detected as an internal control. Plasma EBV loads were quantified by expression of the BamH1W repeats in the EBV genome.

### Synthetic EBV Peptides

Two types of synthetic peptides consisting of 24 HLA class I-restricted peptides and 9 overlapping peptide pools were utilized. All HLA class I-restricted peptides were kindly provided by Professor Alan B. Rickinson (The University of Birmingham, Birmingham, United Kingdom) and their sequences and identities are listed in Supplementary Table 2. The HLA class-I restricted peptides were diluted into 2 μg/mL in CTL-Test Medium (Cellular Technology Limited, United States) and 100 μl was added to each well in 96-well plate. Fifteen-mer peptides overlapped by 11 amino acids spanning each of the EBV latent proteins, EBNA1, EBNA3A, EBNA3B, EBNA3C and LMP2 and the lytic proteins, BZLF1, BRLF1, BMLF1 and GP350 were purchased from JPT Peptide Technologies, Berlin, Germany. The lyophilized peptide pools of each EBV protein were reconstituted according to manufacturer’s instruction. Final concentration of overlapping peptides was 10 μg/ml per million cells. The HLA class-I restricted peptides were used in the ELISPOT assay and overlapping peptides were used in the flow cytometric analysis.

### ELISPOT Assay

Interferon-gamma (IFN-γ) producing EBV-specific CD8+ T cell responses were quantified in duplicate by ELISPOT assay. Cryopreserved PBMC and HLA class-I restricted peptides were used. The peptide epitopes tested for each individual were listed in Supplementary Table 3. Wells containing phytohemagglutinin (PHA) (1 mg/ml) were used as positive control and wells with only cells were used as a negative control. IFN-γ producing cells were counted by immunospot plate reader (Cellular Technology Limited-ImmunoSpot S5 Macro Analyzer) and expressed in spot-forming cells (SFC)/10^6^ PBMC. Spots were defined as positive when the SFC/10^6^ PBMC was higher than 20. Only positive results were reported in the present study.

### Expansion of T Cells

For better sensitivity of the flow cytometric analysis, the EBV lytic and latent protein-specific T cells were expanded by adding recombinant IL-4 and IL-7 according to a protocol provided by Professor Cliona M Rooney, Baylor College of Medicine, United States (Jones et al., 2010; Gerdemann et al., 2011). In brief, cryopreserved PBMC were thawed and washed with 10% FBS in RPMI 1640. Cells were seeded and incubated with relevant peptide pools, IL-4 and IL-7 at 2 μg/ml, 1000 U/ml and 10 ng/ml (PreproTech) respectively for 5 days at 37°C, 5% CO_2_ in 24-well plate (1 × 10^6^ PBMC/ml). PBMC cultured with IL-4 and IL-7 for 5 days were used as negative control.

### Re-stimulation of T Cells

After expansion of T cells for 5 days, PBMC were rested overnight at 37°C, 5% CO_2_ prior to peptide re-stimulation. PBMC were then re-stimulated with corresponding overlapping peptide pools at 2 μg/ml, together with PE-Cy5-conjugated anti-CD107a monoclonal antibody (BD Biosciences Pharmingen, United States), co-stimulatory reagents containing anti-CD28 monoclonal antibody at 1 μg/ml (BD Pharmingen, United States), anti-CD49d monoclonal antibody at 1 μg/ml (BD Pharmingen, United States) and brefeldin A at 10 μg/ml (BD Biosciences Pharmingen, United States) for 6 h at 37°C, 5% CO_2_. Cells stimulated with 1 μg/ml staphylococcal enterotoxin B (SEB) were used as a positive control whereas unstimulated PBMC were used as negative control. Both positive and a negative controls were treated with the same costimulatory reagents.

### Flow Cytometric Analysis

After incubation for 6 h, the treated cells were washed in PBS and stained with Aqua Blue Dye (Invitrogen, United States) for 20 min for dead cell exclusion. APC-Cy7-conjugated anti-CD3 (BD Pharmingen, United States), PE-Texas Red-conjugated anti-CD4 (Life Technologies, United States) and Pacific Blue-conjugated anti-CD8 (BD Pharmingen, United States) monoclonal antibody were subsequently added for surface marker staining. The stained cells were washed, fixed and permeabilized by BD FACS fixation/ permeabilization kit (BD Bioscience). Subsequently, PBMC were washed and stained intracellularly with FITC-conjugated anti-IFN-γ (BD Pharmingen, United States), PE-conjugated anti-perforin (Abcam, United States BD-48 clone), PE-Cy7-conjugated anti-TNF-α (BD Pharmingen, United States) and allophycocyanin (APC)-conjugated anti-IL-2 monoclonal antibody (BD Pharmingen, United States) for 30 min. Cells were washed and resuspended in 1% paraformaldehyde prior to flow cytometric analysis. Around 10^6^ cells were acquired on FACS LSR-II flow cytometer (BD Biosciences, United States). FACS data was analyzed by FlowJo (Tree Star, United States). The data with lymphocytes and live cells below 20% of the total population were excluded. The combinations of T cell functions were analyzed by the Boolean combination gate in FlowJo software. T cells with more than or equal to 3 functions were defined as PFCs.

#### Isolation of CD3+ T Cells and Cytolysis Assay

CD3+ T cells, which were used as effector cells in this study, were isolated and purified from PBMC of 3 IM patients by negative selection using Magnetic Beads Sorting Kit (Pan T cell isolation kit, Miltenyi, Germany). The purity of isolated CD3+ T cells was determined by staining with anti-CD3 monoclonal antibody, followed by analysis with BD LSRII flow cytometer. Highly purified CD3+ T cells (>95%) were obtained after sorting. Autologous LCLs (target cells) were stained with 5- and 6-carboxyfluorescein diascetate succinimidyl ester (CFSE) and incubated in 10% FBS/RPMI at 10^6^ cells/ml at 37°C, 5% CO_2_. The purified and stimulated CD3+ T cells (effector cells) were co-cultured with the target cells at a ratio 10:1 for 4 h at 37°C, 5% CO2 and stained with propidium iodide (PI) (Life Technologies). Cells were analyzed by flow cytometry. Percentage of PI positive cells within the CFSE positive population was obtained. Cultures with either autologous LCLs or isolated CD3+ T cells alone served as negative controls.

### Statistical Analysis

Comparisons of EBV loads in PBMC and plasma amongst the longitudinal samples of IM or AS subjects across the study time points were performed using repeated measure ANOVA. Bonferroni’s correction was applied. *P*-value < 0.0001 was regarded as statistically significant. Comparisons between groups of T cell responses across the study time points were performed by Kruskal–Wallis test and Mann–Whitney Test. *P*-value < 0.05 was regarded as statistically significant. Prism 6 (GraphPad Software, La Jolla, CA, United States) was used for calculations and illustrations.

## Results

### Magnitude of IFN-γ Secreting CD8+ T Cell Responses Toward EBV Lytic Peptides Decreased but Those Toward Latent Peptides Increased Over Time in Both IM and AS Individuals

**FIGURE 1:**
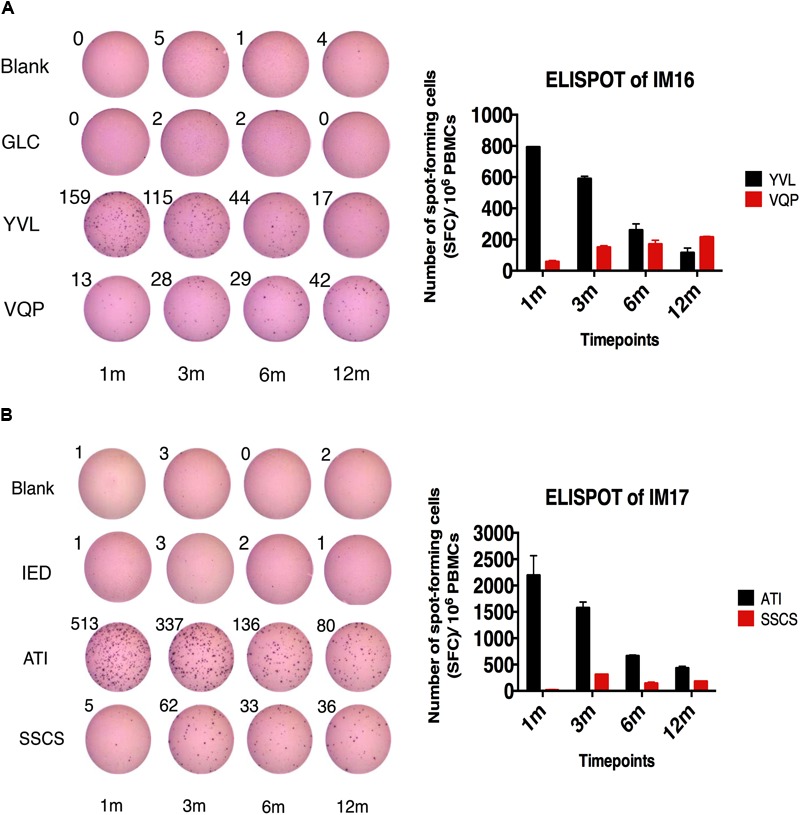
IFN-γ producing EBV-specific CD8+ T cell responses in IM16 and IM 17. **(A)** IM16 cells collected at 0, 1, 3, 6, and 12 months post-diagnosis (0, 1, 3, 6, and 12 m) were stimulated with 7 different peptides (TLD, VLK, GLC, YVL, SLR, CLG, and VQP) in duplicate wells. IM16 reacted to only two of the peptides. GLC was shown as representative negative control. **(B)** IM17 cells collected at 0, 1, 3, 6, and 12 months post-diagnosis (0, 1, 3, 6, and 12 m) were stimulated with 5 different peptides (ATI, SSCS, AVF, IED, and SEN) in duplicate wells. IM17 reacted to only two of the peptides. IED was shown as representative negative control. Wells with cells only (blank) were also shown as negative controls. The numbers of spots were shown above the wells. Positive results in histogram were expressed in the mean ± SEM of numbers of spot-forming cells (SFC) per million PBMC.

We measured the magnitude of EBV-specific CD8+ T cell responses toward EBV lytic and latent peptides in both IM and AS individuals by IFN-γ ELISPOT assay. HLA typing of 18 PBMC samples of IM patients (IM1–18) and 12 AS individuals (AS 1–12) were performed (Supplementary Table 3). HLA-class I specific EBV lytic and latent peptides were used to stimulate the PBMC of HLA-class I matched individuals (Supplementary Tables 2, 3). The number of spot-forming cells (SFC) per million PBMC of the IM and AS individuals is shown in **Table 1**. Trend in changes of the EBV antigen-specific T cell response was deduced by comparing the SFC per million PBMC at the earliest time point to that at the longest time point for each study subject (**Table 1**). Difference in the magnitude of the T cell responses less than or equal to 20 SFC per million PBMC was defined as a stable trend whereas that greater than 20 SFC per million PBMC was defined as an increasing or decreasing trend. As indicated in **Table 1**, the magnitude of IFN-γ secreting CD8+ T cell responses toward EBV lytic peptides tended to decrease over time from day 0 to 12 months whereas those toward latent peptides tended to increase over time in the majority of IM and AS subjects. The ELISPOT data of 2 representative cases, IM16 and IM17, were shown in **Figure 1**. PBMC of IM16 (HLA-type: A2, A26, B46 and cw1) collected at 1, 3, 6, and 12 months post-diagnosis were stimulated by 7 peptide epitopes (TLD, VLK, GLC, YVL, SLR, CLG, VQP). Prominent IFN-γ secreting CD8+ T cell responses against an immediate lytic cycle protein BRLF1-derived peptide (YVL) and a latent cycle protein EBNA3A-derived peptide (VQP) were observed (**Figure 1A**). YVL stimulated 793 SFC at 1 month and decreased to 115 SFC at 12 months. In contrast, VQP stimulated 58 SFC at 1 month and increased to 215 SFC at 12 months. IM17 (HLA-type: A1, A11, B4001, B13, Cw7 and Cw10) showed similar trend of T cell responses (**Figure 1B**). Response against ATI, the A11-restricted BRLF1-derived peptide, was found to decrease from 2193 to 433 SFC whereas the response against SSCS, the LMP2A-derived peptide, was found to increase from 15 to 180 SFC from 1 to 12 months (**Figure 1B**). The decreasing trend of IFN-γ secreting CD8+ T cell responses toward lytic peptides and increasing trend of responses toward latent peptides were observed in the majority of the IM and AS subjects (**Table 1**). No significant difference between the responses in IM and AS individuals was detected.

**Table 1:** Table showing the T cell responses against HLA-class I restricted EBV lytic and latent peptides determined by ELISPOT.

Status	Peptide	Protein	Type	D0	D7	1 m	3 m	6 m	12 m	Trend
IM1	SSCS	LMP2A	Latent	_	3	18	35	45	55	Increasing
IM1	ATI	BRLF1	Lytic	_	413	268	65	65	43	Decreasing
IM1	SEN	BZLF1	Lytic	_	50	23	18	35	20	Decreasing
IM2	SSCS	LMP2A	Latent	_	_	_	165	_	_	_
IM3	VSF	EBNA3B	Latent	1610	_	_	2065	_	_	Increasing
IM4	RPP	EBNA3A	Latent	1823	393	603	525	_	298	Decreasing
IM4	RPQG	BMRF1	Lytic	533	138	310	293	_	93	Decreasing
IM5	YVL	BRLF1	Lytic	_	_	813	893	328	_	Decreasing
IM5	SSCS	LMP2A	Latent	_	_	23	30	123	_	Increasing
IM5	ATI	BRLF1	Lytic	_	_	453	370	163	_	Decreasing
IM6	VSF	EBNA3B	Latent	_	_	_	785	1380	1753	Increasing
IM6	SLR	EBNALP	Latent	_	_	_	470	365	470	Stable
IM7	EPL	BZLF1	Lytic	_	505	_	1008	_	805	Increasing
IM8	SEN	BZLF1	Lytic	_	3423	2260	1133	_	1025	Decreasing
IM9	GLC	BMLF1	Lytic	_	333	_	_	_	93	Decreasing
IM9	AVF	EBNA3B	Latent	_	985	_	_	_	8	Decreasing
IM9	VQP	EBNA3A	Latent	_	18	_	_	_	123	Increasing
IM9	SSCS	LMP2A	Latent	_	33	_	90	_	_	Increasing
IM10	IED	LMP2A	Latent	_	63	_	1488	413	223	Increasing
IM10	SEN	BZLF1	Lytic	_	488	_	305	245	28	Decreasing
IM11	SSCS	LMP2A	Latent	_	0	113	_	298	_	Increasing
IM11	VSF	EBNA3B	Latent	_	383	563	_	383	_	Stable
IM11	ATI	BRLF1	Lytic	_	78	80	_	75	_	Stable
IM12	VSF	EBNA3B	Latent	_	775	_	_	393	283	Decreasing
IM13	VLK	EBNA1	Latent	_	30	_	83	_	258	Increasing
IM13	SLR	EBNALP	Latent	_	75	_	78	_	88	Stable
IM14	YVL	BRLF1	Lytic	_	_	133	90	25	28	Decreasing
IM14	AVF	EBNA3B	Latent	_	_	8	548	163	178	Increasing
IM14	IED	LMP2A	Latent	_	_	43	525	385	403	Increasing
IM15	ATI	BRLF1	Lytic	_	_	18	53	60	73	Increasing
IM15	VSF	EBNA3B	Latent	_	_	263	225	103	155	Decreasing
IM15	SSCS	LMP2A	Latent	_	_	13	73	78	93	Increasing
IM16	YVL	BRLF1	Lytic	_	_	793	590	260	115	Decreasing
IM16	VQP	EBNA3A	Latent	_	_	58	150	170	215	Increasing
IM17	SSCS	LMP2A	Latent	_	_	15	310	143	180	Increasing
IM17	ATI	BRLF1	Lytic	_	_	2193	1575	663	433	Decreasing
IM17	SEN	BZLF1	Lytic	_	_	65	90	48	20	Decreasing
IM18	VLK	EBNA1	Latent	_	_	_	375	180	370	Stable
IM18	SSCS	LMP2A	Latent	_	_	_	128	15	150	Increasing
AS6	VSF	EBNA3B	Latent	_	_	305	530	_	_	Increasing
AS7	AVF	EBNA3B	Latent	_	_	0	20	_	98	Increasing
AS7	SSCS	LMP2A	Latent	_	_	90	510	_	223	Increasing
AS7	ATI	BRLF1	Lytic	_	_	213	148	_	28	Deceasing
AS8	SSCS	LMP2A	Latent	_	_	_	45	168	_	Increasing
AS11	AVF	EBNA3B	Latent	_	_	_	185	295	275	Increasing
AS11	SSCS	LMP2A	Latent	_	_	_	23	95	93	Increasing
AS12	VSF	EBNA3B	Latent	_	_	_	185	408	273	Increasing

*Results are expressed as numbers of spot-forming cells (SFC). “–“ represents sample not available.*

### PBMC and Plasma Viral Loads in Both IM and AS Individuals Decreased Over Time

**FIGURE 2:**
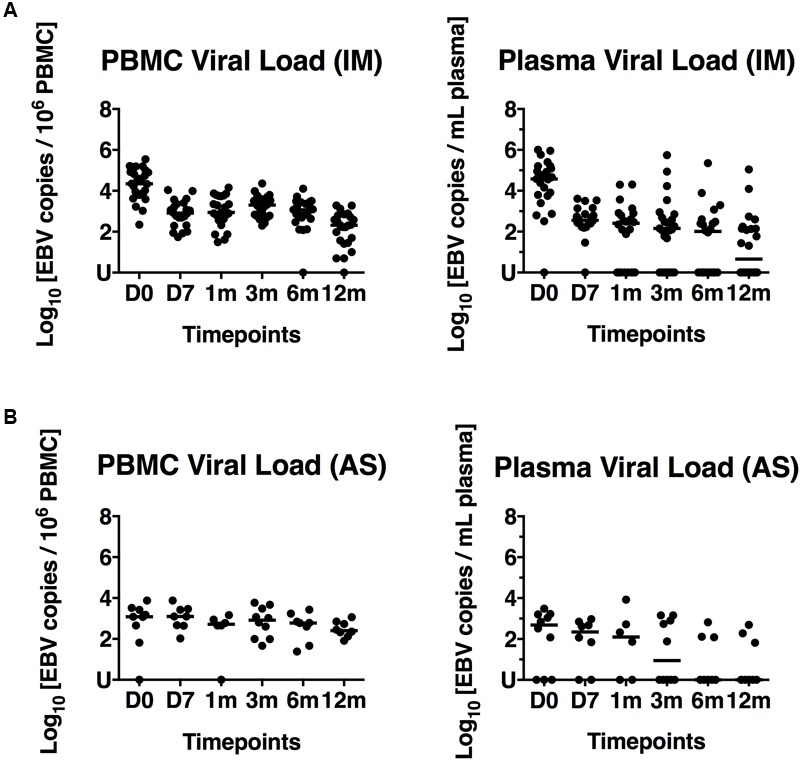
PBMC and plasma viral loads of 29 IM patients and 12 AS individuals. **(A)** EBV loads in PBMC and plasma of IM1-29. **(B)** EBV loads in PBMC and plasma of AS1-12. The spots represent the copy numbers of EBV per million PBMC and copy numbers of EBV per mL plasma in different IM and AS individuals collected at Day 0, 7 and 1, 3, 6, and 12 months post-diagnosis (D0, D7, 1 m, 3 m, 6 m, and 12 m). U, undetectable.

High PBMC viral loads in IM patients during acute infection and substantial decrease of viral loads over time were observed in previous studies (Kimura et al., 1999; Balfour et al., 2005; Cheng et al., 2007). We also determined the viral loads in both PBMC and plasma of 29 IM patients and 12 AS subjects by qPCR. The median PBMC viral load in IM patients decreased from 21878 EBV copies/million PBMC at diagnosis (D0) to 209 copies/million PBMC at 12 months after diagnosis (**Figure 2A**). The median plasma viral loads also decreased from 37154 to 5 copies/mL plasma from day 0 to 12 months (**Figure 2A**). Decreases in PBMC and plasma viral loads were also observed in AS individuals (**Figure 2B**). In both IM and AS subjects, the PBMC and plasma viral loads peaked at diagnosis and declined substantially afterward. The viral loads were the lowest at the end of the study period, indicating an establishment of effective viral control. Repeated measure ANOVA with Bonferroni’s correction was applied for multiple comparisons. Cases which had a complete set of time points (day 0, day 7 and 1, 3, 6, and 12 months) were evaluated by the program. The results showed that the PBMC (*n* = 9) and plasma viral loads (*n* = 7) significantly decreased over time in the IM samples with values [*F*(5,40) = 12.76, *P* < 0.0001] and [*F*(5,30) = 13.23, *P* < 0.0001], respectively. In addition, the PBMC and plasma viral loads at day 7 and 1, 3, 6, and 12 months were significantly lower than those at day 0 (Supplementary Figure 1).

### Kinetics of Development of CD4+ and CD8+ T Cell Responses to EBV Lytic and Latent Cycle Antigens After Primary Infection

Next, we carried out a detailed characterization of the development of CD4+ and CD8+ T cell responses to several immunodominant EBV lytic and latent antigens in IM patients from primary infection to long term persistence by multi-color flow cytometric assays. Due to the low frequencies of EBV-specific T cells, an expansion of T cells was necessary for the accurate analysis of the subtle changes of functional T cell subsets in the IM patients during different stages of infection (Jennes et al., 2002; Jones et al., 2010). We adopted a T cell expansion protocol provided by Prof. Cliona M Rooney (Baylor College of Medicine, United States) in order to increase the sensitivity of our flow cytometric assay (Gerdemann et al., 2011). Such expansion protocol using IL4 and IL7 had been used in different T cell studies and found to maintain the survival of most of the cytokine secreting T cells in similar magnitude (Vella et al., 1997, 1998; Gerdemann et al., 2011; Shmarov et al., 2016). To confirm the validity of the flow cytometric assay, we stimulated the PBMC with EBV overlapping peptide pools (EBNA1, EBNA-3A, -3B, -3C and BZLF1) in an IM patient and an EBV-seronegative donor. EBV-specific T cells were clearly detected in the IM patient but not in the seronegative donor except for the pre-loaded perforin in a small proportion of T cells (Supplementary Figure 2). In addition, the increasing trend of latent antigen-specific IFN-γ secreting CD8+ T cell responses and decreasing trend of lytic antigen-specific responses detected by ELISPOT could also be replicated by this flow cytometric assay (Supplementary Figure 3).

**FIGURE 3:**
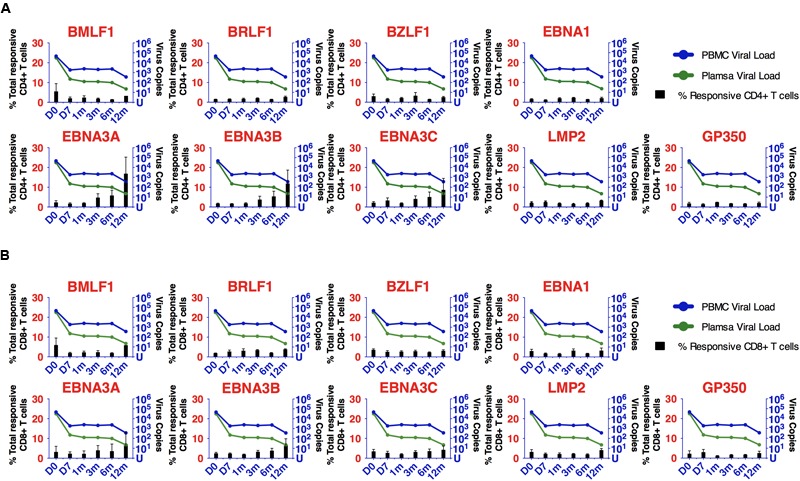
Kinetics of development of CD4+ and CD8+ T cell responses to EBV lytic and latent cycle antigens after primary infection. **(A)** Kinetic changes of the percentages of EBV-specific CD4+ T cells and EBV loads. **(B)** Kinetic changes of the percentages of EBV-specific CD8+ T cells and EBV loads. T cells were expanded by overlapping peptide pools for 5 days and re-stimulated by the corresponding peptide pools for 6 h before flow cytometric assays. Each bar represents the mean percentages of EBV-specific T cells in the 11 IM patients (IM 19–29) at different time points. The corresponding viral loads in PBMC (blue curve) and plasma (green curve) at different time points are shown. Each dot in the line curve represents the mean viral load of the 11 IM patients.

We proceeded to analyze the EBV-specific T cell responses of 11 longitudinally followed IM patients (IM19-29) toward 4 lytic and 5 latent overlapping EBV peptides. The magnitude of responses was measured as the frequency of responsive T cells which presented at least one function (either IFN-γ, perforin, TNF-α, IL-2, or CD107a) after stimulation by the overlapping peptide pools. No discernible differences in the magnitude and quality of T cell responses were found among the IM patients of different age groups. The overall magnitude of responses of CD4+ T cells to overlapping peptide pools of lytic and latent cycle proteins was similar to that of CD8+ T cells (**Figure 3**). At diagnosis, all EBV lytic peptide pools could stimulate both CD4+ and CD8+ T cell responses, presenting a broad response spectrum. Remarkably, the frequency of EBNA3A, 3B and 3C-specific CD4+ and CD8+ T cells clearly increased over time and dominated at 1 year after diagnosis. In contrast, responses toward other EBV lytic and latent peptides were relatively stable throughout the period of our study (**Figure 3**).

### Diversity of CD4+ and CD8+ Polyfunctional T Cells (PFC) Responses Increased Over Time

**FIGURE 4:**
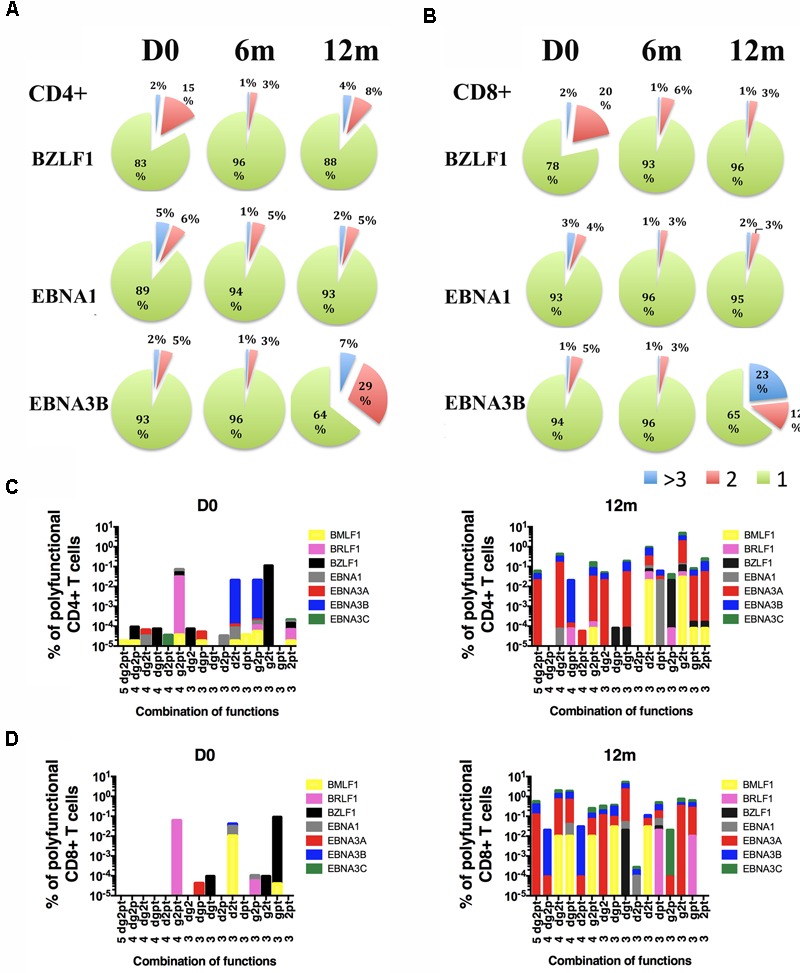
Longitudinal functional profiles of EBV-specific T cells in one representative IM patient (IM25). The pie charts represent the proportions of **(A)** CD4+ T cells and **(B)** CD8+ T cells with single (green), double (red), triple or more (blue) functions upon stimulation with BZLF1, EBNA3B, and EBNA1 overlapping peptide pools. T cells were expanded with 4 lytic and 5 latent antigens overlapping peptides pools for 5 days and re-stimulated by the corresponding peptide pools for 6 h before flow cytometric assays. Flow cytometric assays were performed at an early time point ranged from D0 to 3 m, a middle time point ranged from 3 to 6 m and a late time point ranged from 6 to 12 m. D0, at diagnosis; 6 m, 6 months after diagnosis; 12 m, 1 year after diagnosis. The percentages of responsive **(C)** CD4+ and **(D)** CD8+ T cells with different combinations of functions upon stimulation by BMLF1, BRLF1, BZLF1, EBNA1, EBNA3A, EBNA3B and EBNA3C at diagnosis (D0) and 1 year after diagnosis (12 m). d, CD107a; g, Interferon-gamma; 2, Interleukin-2; p, Perforin; t, Tumor-necrosis factor-alpha.

Polyfunctional T cells, which execute multiple functions upon stimulation by a specific antigen, were reported to elicit stronger immune responses (Betts et al., 2006; Almeida et al., 2007; Smith et al., 2009). We examined the evolution of the magnitude and functionality of lytic and latent cycle antigen-specific CD4+ and CD8+ T cells. The functional profiles of CD4+ and CD8+ PFC (with three or more functions) of a representative case, IM 25, are shown in **Figure 4**. Low percentages of lytic and latent antigen-specific CD4+ and CD8+ responsive T cells with 2 or more functions appeared at diagnosis. The CD4+ and CD8+ responsive T cells against the latent antigen EBNA3B increased dramatically over time. Particularly, the frequency of EBNA3B-specific CD8+ PFC increased from approximately 1% at diagnosis to 30% of total antigen-reactive CD8+ T cells at 12 months after diagnosis. In contrast, BZLF1-responsive cells decreased over time and EBNA1- responsive cells remained stable across the time points from day 0 to 12 months (**Figures 4A,B**). At day 0, most of the CD4+ and CD8+ PFC only displayed a restricted number of combinations of functions. Very low percentages of 4-functional CD4+ PFC (IFNγ+/IL-2+/perforin+/TNF-α+) and 3-functional CD8+ PFC (CD107a+/IL-2+/TNF-α+) were detected (**Figures 4A,B**). At later time points, these CD4+ and CD8+ PFC became more highly functional as 4- and 5-functional T cells started to emerge. Interestingly, a dramatic increase in the diversity of functional subsets of both CD4+ and CD8+ T cells was observed (**Figures 4C,D**). Such diverse functional subsets were found in 8 out of the 11 IM cases whereas the remaining 3 cases showed a constant level or slightly decreased variety of combination of functions in the later time points (Supplementary Figure 4).

### Emergence of CD4+ and CD8+ Polyfunctional T Cell Responses Toward EBV Lytic and Latent Antigens

**FIGURE 5:**
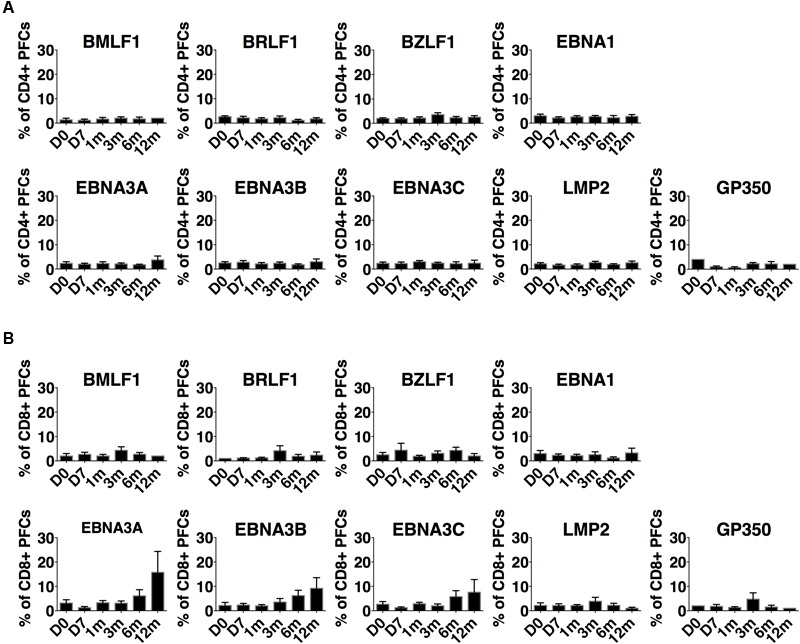
Development of EBV lytic and latent antigen-specific polyfunctional T cells from acute primary infection stage to long-term persistence. **(A)** CD4+ T cells and **(B)** CD8+ T cells in 11 IM patients. T cells were expanded by overlapping peptide pools for 5 days and re-stimulated by the corresponding peptide pools for 6 h before flow cytometric assays. Each bar represents the mean ± SEM percentages of polyfunctional T cells (three or more functions after stimulation) out of total EBV-specific T cells upon stimulation with a specific latent overlapping peptide pool.

We summarized the development of EBV-specific CD4+ and CD8+ PFC for all IM patients (**Figure 5**). EBV lytic and latent cycle antigen-specific CD4+ PFC (with three or more functions) existed at diagnosis but the mean frequencies were less than 3% of total responsive CD4+ T cells (**Figure 5A**). The mean frequencies of CD4+ PFC against lytic and latent cycle antigens did not change significantly over time from primary infection to long term persistent stage. The abilities of EBV lytic and latent cycle antigen-specific CD8+ T cells to generate PFC were relatively stronger when compared with CD4+ cells and appeared to change over time (**Figure 5B**). During acute infection, the mean frequencies of EBV-specific CD8+ PFC were less than 3% of the total responsive CD8+ T cells after stimulation. The percentages of CD8+ PFC against early lytic antigen, BMLF1, BRLF1 and BZLF1, increased slightly whereas those of CD8+ PFC against the latent antigens, EBNA3A, 3B and 3C, increased significantly over time. Other subdominant antigens such as EBNA1, LMP2 and GP350 did not stimulate any significant changes in the proportion of PFC. Although the PFC responses were induced by the immunodominant latent antigens derived from EBNA3A-C in most of the IM patients (**Figure 5**), it should be noted that PFC responses could also be induced by antigens derived from immediately early (BZLF1 and BRLF1) and early (BMLF1) EBV proteins in some individuals (Refer to **Figures 4, 5** and Supplementary Figure 4). Taken together, our data suggested that the CD4+ and CD8+ T cells specific to the immunodominant EBV proteins, including BZLF1, BRLF1, BMLF1, EBNA-3A, -3B, and -3C, became highly functional during the long term persistent phase.

### Increased Cytotoxicity of T Cells Against Autologous LCL in IM Patients Over Time

Cytotoxicity assay was carried out to examine whether increased polyfunctionality is accompanied by enhanced cytotoxicity of EBV-specific T cells. T cells were stimulated and expanded by overlapping peptide pools from the immunodominant EBNA3A or 3B protein of each donor. The CD3+ T cells were then purified by negative selection using Miltenyl Magnetic Beads Cell Sorting system. The purity of the T cells was on average greater than 95%, and the viability of T cells after sorting did not change significantly.

**FIGURE 6:**
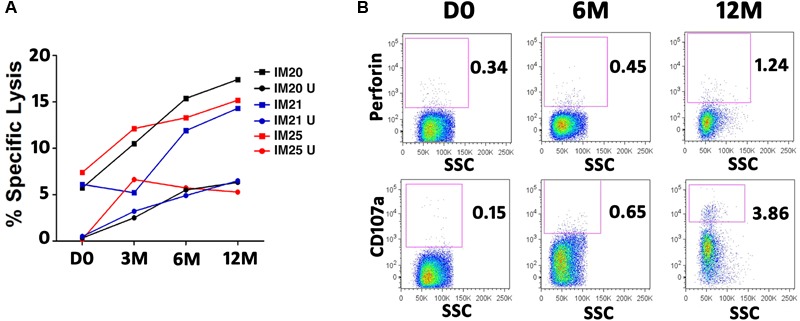
Cytotoxicity of EBNA3-specific T cells against autologous LCLs in three IM patients. T cells were expanded for 5 days in the presence of overlapping peptide pools of EBNA3A or EBNA3B. Autologous B-LCL (target cells) were stained with CFSE and co-cultured with activated purified T cells (effector cells) for 4 h at an Effector/Target ratio of 10:1. Dead cells were discriminated from live cells by propidium iodide (PI). Autologous LCLs were killed by T cells. The percentage of specific lysis is calculated by subtracting the background (LCL co-cultured with unstimulated T cells). **(A)** Cytotoxicity of EBNA3-specific T cells at different time points in 3 IM patients. PBMC of IM20 and IM21 and IM25 patients were stimulated and expanded with overlapping peptide pools of EBNA3A and EBNA3B, respectively. U, LCL co-cultured with unstimulated T cells. **(B)** Development of Perforin producing T cells (upper row) and CD107a positive T cells (lower row) in IM25.

The cytotoxicity of T cells against autologous LCL, after stimulation with EBNA3 overlapping peptide pools, was assessed longitudinally for three IM patients (**Figure 6A**). The cytotoxicity of T cells against LCL increased over time. These results were consistent with elevated frequencies of perforin- and CD107a-expressing T cells (**Figure 6B**). Cytotoxic potential was further enhanced by stimulating the T cells with EBNA3 overlapping peptide pools for 5 days prior to setting up the lysis assay, concurrent with increasing polyfunctionality, perforin production, and degranulation (**Figure 6B**).

## Discussion

Identification of polyfunctional CD8+ T cells in the immune control of viral infection had been reported (Betts et al., 2006; Almeida et al., 2007; Smith et al., 2009). However, most of these studies focused on assessing CD8+ T cell responses to human immunodeficiency virus (HIV) and their mechanisms in killing. Much less is known about the role of PFC responses in the control of stable persistent DNA virus such as EBV. Here, we describe a comprehensive study in which the development of EBV-specific CD4+ and CD8+ T cells, as well as their magnitude and polyfunctionality were monitored from primary infection to long-term persistence in two cohorts of children with infectious mononucleosis (IM) and asymptomatic (AS) primary EBV infection, respectively.

The IFN-γ secreting CD8+ T cells were characterized as the major population of T cells participating in viral control (Callan et al., 1996; Shi and Lutz, 2002). IFN-γ ELISPOT assay became a common quantitative detection method of virus-specific CD8+ cells (Larsson et al., 1999; Yang et al., 2000). A previous study had demonstrated the diagnostic potential of IFN-γ and IL-2 ELISPOT assays in distinguishing active and latent tuberculosis in children infected with *Mycobacterium tuberculosis* (Chiappini et al., 2012). In the present study, the kinetics of IFN-γ producing CD8+ T cell responses in IM patients and AS subjects was first assessed by ELISPOT assay. The decreasing pattern of IFN-γ producing CD8+ T cell responses toward EBV lytic peptides and increasing pattern of T cell responses toward EBV latent peptides were identified over time in both IM and AS subjects. There was no discernible difference in the trend of evolution of T cell responses in these two study cohorts. However, this widely accepted IFN-γELISPOT assay using HLA-type restricted epitopes to detect EBV antigen-specific T cells could not simultaneously assess the CD4+ cell responses and determine the expression of other functional cytokines (Casazza et al., 2006; Scherrenburg et al., 2008). The difficulties in choosing the specific EBV peptides across a wide range of HLA alleles also restricted the panel of antigen-specific cells to be tested which could result in missing of potentially important information (Supplementary Tables 2, 3).

Accumulating evidence indicated that the “quality” of T cell responses should be assessed by functionality (Seder et al., 2008). The more functions the T cells possess, the more robust the immune protection they can provide (Makedonas et al., 2010). We have also shown that long term carriers could generate robust PFC responses against lytic and latent EBV antigens with the greatest responses toward the immunodominant epitopes of the EBNA3 proteins (Ning et al., 2011). However, the time of emergence of PFC responses against EBV lytic or latent antigens, differences between CD4+ and CD8+ T cell responses, and concurrent EBV loads in PBMC and plasma from acute to chronic stage of infection are not clearly understood. To address these questions, we performed a longitudinal study to assess the development of PFC responses to EBV in 11 IM patients. We assessed five functional parameters: IFN-γ, TNF-α, IL-2, perforin, and CD107a, all of which are typical functions of T cells that were reported in previous studies (Harari et al., 2006; Pantaleo and Harari, 2006; Precopio et al., 2007; Makedonas et al., 2010). T cells with three or more functions after stimulation are defined as PFC. EBV-specific lytic and latent antigen-derived overlapping peptides were used to stimulate both CD4+ and CD8+ T cell responses. Similar trend of response of IFN-γ specific CD8+ cells was observed as that detected by ELISPOT assay, validating the reliability of data generated by both methods (Supplementary Figure 3). In the IM patients, EBV-specific CD4+ and CD8+ PFC were detectable at diagnosis toward lytic and latent cycle antigens. These CD4+ and CD8+ PFC only displayed a restricted combination of functions, such as the 4- functional CD4+ PFC (IFNγ+/IL-2+/perforin+/TNF-α+) and the 3-functional CD8+ PFC (CD107a+/IL-2+/TNF-α+) (refer to **Figure 4**). At later time points, these CD4+ and CD8+ PFC became more highly functional as 4- and 5-functional T cells started to emerge. The development of PFC responses toward different specific peptides varied among the individuals. In most of the IM patients, the PFC responses were induced by the immunodominant antigens derived from EBNA3A-C (**Figure 5**). Interestingly, in some of the individuals, antigens derived from immediately early (BZLF1 and BRLF1) and early (BMLF1) EBV proteins induced the highest proportion of CD8+ as well as CD4+ PFCs (refer to **Figures 4, 5** and Supplementary Figure 4). In addition, low frequencies of EBNA1-specific CD4+ T cells were detected at both diagnosis and 6 and 12 months in some IM patients (Supplementary Figure 4), in contrast to the findings of previous studies in which EBNA1-specific CD4+ T cells were not detected until months after the initial diagnosis (Long et al., 2013; Rickinson et al., 2014). Since EBNA1-specific effector T cells might be important in the control of EBV-positive lymphoproliferative diseases (Jones et al., 2010), it is interesting to follow whether the proportion of EBNA1-responsive PFC would increase at longer time points in the future. Diverse combinations of three or more functions of both antigen-specific CD4+ and CD8+ T cells were shown to emerge at 6–12 months, suggesting a potential co-operative relationship between the CD4+ and CD8+ PFC in the long term viral control (**Figures 4C,D** and Supplementary Figure 4). Presence of CD107a+ and perforin-releasing cytotoxic CD4+ PFC was demonstrated in this study (**Figure 4C**). Similarly, a rare population of CD4+ cytotoxic T cells was previously identified in the patients with chronic viral infection of CMV (Casazza et al., 2006). The presence of cytotoxic CD4+ PFC indicates that CD4+ T cells may also play a direct role in the control of persistent EBV infection. The clinical preparation of virus-specific T cells for the control of post-transplant lymphoproliferative disorders was shown to require the presence of CD4+ T cells (Gerdemann et al., 2011; Linnerbauer et al., 2014). In fact, CD4+ PFC was found to be molecularly distinct from IFN-γ mono-functional CD4+ T cells and associate with favorable clinical outcomes in patients with *Plasmodium falciparum* infection (Burel et al., 2017). Taken together, our data clearly demonstrated the development and maturation of both CD4+ and CD8+ PFC from early to long term infection stages in IM patients.

Decreasing patterns of EBV loads in PBMC and plasma were illustrated in both IM and AS subjects (**Figure 2**), indicating that both study subjects were capable of generating sufficient T-cell immunity for viral control. Moreover, the viral loads *per se* could not explain the development of IM symptoms in some individuals, although elevated viral loads were observed in both IM and AS individuals during acute EBV infection (Silins et al., 2001). Manifestation of the self-limiting immunopathological conditions arising in IM patients might be related to the inadequate NK cell cytotoxicity toward infected cells during acute infection (Rickinson et al., 2014). In both IM and AS subjects, plasma loads declined dramatically to low or undetected levels, yet, PBMC viral loads showed a slower decline (**Figure 2**). The sharp decrease in plasma loads was probably due to the initial control by NK cell and the early lytic cycle antigen-specific CD8+ T cell responses. In contrast, the slow decline of PBMC was due to the gradual emergence of both lytic and latent antigen-specific polyfunctional CD4+ and CD8+ T cell responses at about 6 months. Indeed, a natural decay of PBMC viral loads in the early stage of infection independent of EBV-specific T-cell immunity was previously proposed (Hadinoto et al., 2008). The viral control thereafter was probably mediated by the development and strengthening of PFC responses toward the immunodominant antigens derived from BZLF1, BRLF1, BMLF1, EBNA3A, 3B and 3C proteins from about 6 to 12 months post-infection. The sustained decline of EBV loads during this period correlated well with the emergence of T cells capable of presenting multiple functions. To further assess whether increased PFC responses correlated to viral control in the IM patients, a cytotoxic assay was performed. We stimulated the T cells of three IM patients with EBNA3A or EBNA3B peptide pools and found a steady increase in cytotoxicity of T cells against autologous LCL over time (**Figure 6**). The increasing proportions of EBNA3A- or EBNA3B-specific PFC correlated with the enhanced cytotoxic capacities, supporting that effective viral control was achieved by the capability to generate highly functional T cells.

## Conclusion

EBV antigen-specific CD4+ and CD8+ PFC responses emerge during the first year of primary EBV infection, with greatest responses toward immunodominant epitopes in both lytic (BZLF1, BRLF1 and BMLF1) and latent (EBNA3A-C) proteins, correlating to steady decline in PBMC and plasma viral loads.

## Ethics Statement

This study was carried out in accordance with the recommendations of “Informed Consent of Human Subjects of Research, Institutional Review Board of the University of Hong Kong” with written informed consent from all subjects. All subjects gave written informed consent in accordance with the Declaration of Helsinki. The protocol was approved by the “Institutional Review Board of the University of Hong Kong.”

## Author Contributions

JL, KH, RN, and XX performed the experiments, data analysis, and interpretation. JL, KH, and AC wrote the manuscript. AC coordinated the project and recruited and followed all patients. KC performed the serological assays for all patients.

## Conflict of Interest Statement

The authors declare that the research was conducted in the absence of any commercial or financial relationships that could be construed as a potential conflict of interest.
