# Supplementary material for: Emergence of CD4+ and CD8+ Polyfunctional T Cell Responses Against Immunodominant Lytic and Latent EBV Antigens in Children With Primary EBV Infection
Source: Front Microbiol. 2018 Mar 7;9:416. doi: 10.3389/fmicb.2018.00416 (PMC5863510; doi:10.3389/fmicb.2018.00416)
Supplement: Supplementary file 1 [file Table_1.PDF]

Supplementary Table 1. Basic characteristics of peripheral blood donors. The age, gender, and serology tests with EBV-specific antibodies (anti-viral capsid antigen (VCA)-IgM, anti-VCA-IgG, and anti-EBNA-IgG) for 29 IM patients and 12 AS subjects are shown. EBNA ACIF is a test of anti-EBNA IgG. The pattern of EBV-specific antibodies indicated that IM patients had a very recent EBV primary infection and that AS subjects had primary EBV infection within a period of about 6 months.

Subject ID	*Age at diagnosis (year)*	*Gender*	*VCA-IgM*	*VCA-IgG*	*EBNA ACIF*
IM 1	13.5	M	Equivocal	>640	Positive
IM 2	8.8	M	Positive	>640	Positive
IM 3	2.4	M	Positive	>640	Negative
IM 4	5.3	M	Positive	>640	Negative
IM 5	9.7	F	Positive	>640	Negative
IM 6	4.7	M	Positive	>640	Negative
IM 7	2.5	M	Positive	>640	Negative
IM 8	7.6	M	Positive	>640	Negative
IM 9	6.8	F	Positive	>640	Negative
IM 10	3.3	M	Positive	>640	Negative
IM 11	3.2	M	Positive	640	Negative
IM 12	2.2	M	Positive	>640	Negative
IM 13	4	M	Positive	>640	Negative
IM 14	15.3	F	Equivocal	>640	Negative
IM 15	12.8	M	Positive	>640	Negative
IM 16	8.9	F	Positive	>640	Positive
IM 17	17.9	F	Positive	>640	Negative
IM 18	10.2	M	Positive	>640	Negative
IM 19	3.9	M	Positive	>640	Negative
IM 20	1.5	M	Positive	>640	Negative
IM 21	2.3	M	Negative	640	Negative
IM 22	3.5	M	Positive	>640	Negative
IM 23	6.3	M	Positive	>640	Negative
IM 24	5.8	F	Positive	>640	Negative
IM 25	6.3	M	Positive	640	Negative
IM 26	15.2	M	Positive	>640	Negative
IM 27	11	F	Positive	>640	Negative
IM 28	15.2	F	Positive	>640	Negative
IM 29	7.4	M	Positive	>640	Negative

Subject ID	*Age at diagnosis (year)*	*Gender*	*VCA-IgM*	*VCA-IgG*	*EBNA ACIF*
AS 1	3.3	M	Negative	40	Negative
AS 2	8.7	M	Positive	>640	Positive
AS 3	1.1	M	Negative	>640	Negative
AS 4	4.5	F	Positive	>640	Positive
AS 5	10.5	M	Positive	>640	Negative
AS 6	2.4	F	Positive	>640	Negative
AS 7	1.8	M	Positive	160	Negative
AS 8	13.3	F	Positive	>640	Positive
AS 9	3.4	F	Positive	>640	Positive
AS 10	2.8	M	Negative	>640	Negative
AS 11	8.9	F	Positive	>640	Positive
AS 12	3.1	M	Positive	>640	Positive
