# Supplementary material for: Emergence of CD4+ and CD8+ Polyfunctional T Cell Responses Against Immunodominant Lytic and Latent EBV Antigens in Children With Primary EBV Infection
Source: Front Microbiol. 2018 Mar 7;9:416. doi: 10.3389/fmicb.2018.00416 (PMC5863510; doi:10.3389/fmicb.2018.00416)
Supplement: Supplementary file 2 [file Table_2.PDF]

Supplementary Table 2. Human leukocyte antigen (HLA)-class-I-restricted peptides from EBV lytic or latent proteins used in ELISPOT assays.

HLA Panel	HLA type	Peptide name	Amino Acid sequence	Protein	Residues
**A2**	A0201	**TLD**	**TLD**YKPLSV	BMRF1	208-216
	A0201	**GLC**	**GLC**TLVAML	BMLF1	280-288
	A0201	**YVL**	**YVL**DHLIVV	BRLF1	109-117
	A0203	**VLK**	**VLK**DAIKDL	EBNA1	574-582
	A0203	**SLR**	**SLR**EWLLRI	EBNA-LP	284-292
	A2	**CLG**	**CLG**GLLTMV	LMP2 A	426-434
**A11**	A1101	**ATI**	**ATI**GTAMYK	BRLF1	134-142
	A1101	**SSC**	**SSC**SSCPLSKI	LMP2A	340-350
	A1101	**AVF**	**AVF**DRKSDAK	EBNA3B	399-408
**A24**	A24	**TYP**	**TYP**VLEEMF	BRLF1	198-206
	A2402	**IACP**	**IACP**IVMRYVLDHLI	BRLF1	101-115
	A25	**VMS**	**VMS**NTLLSAW	LMP2	442-451
	A2402	**TYG**	**TYG**PVFMCL	LMP2A	419-427
	A2301	**PYL**	**PYL**FWLAAI	LMP2	131-139
	A24	**RYS**	**RYS**IFFDY	EBNA3A	246-253
**B7**	B7	**RPQG**	**RPQG**GSRPEFVKL	BMRF1	116-128
	B7	**RPP**	**RPP**IFIRRL	EBNA3A	379-387
	B7	**RPQ**	**RPQ**KRPSCI	EBNA1	72-80
**B35**	B35	**EPL**	**EPL**PQGQLTAY	BZLF1	54-63
	B35	**HPV**	**HPV**GEADYFEY	EBNA1	407-417
**B4001**	B4001	**SEN**	**SEN**DRLRLL	BZLF1	209-217
	B4001	**IED**	**IED**PPFNSL	LMP2A	200-208
**B46**	B4601	**VQP**	**VQP**PQLTLQV	EBNA3A	617-625
**B58**	B5801	**VSF**	**VSF**IEFVGW	EBNA3B	279-287
