# Supplementary material for: Emergence of CD4+ and CD8+ Polyfunctional T Cell Responses Against Immunodominant Lytic and Latent EBV Antigens in Children With Primary EBV Infection
Source: Front Microbiol. 2018 Mar 7;9:416. doi: 10.3389/fmicb.2018.00416 (PMC5863510; doi:10.3389/fmicb.2018.00416)
Supplement: Supplementary file 3 [file Table_3.PDF]

**Supplementary Table 3. Subject characteristics and EBV peptides used in ELISPOT and flow cytometric assays.**

| Subject ID | HLA type | ELISPOT performed | Flow cytometry performed | EBV Peptide used |
|---|---|---|---|---|
| IM1 | A11, B4001, B46, CW1, CW7 | Yes | No | ATI, SSCS, AVF, IED, SEN, VQP |
| IM2 | A11, B4001, B27, CW7, CW12 | Yes | No | ATI, SSCS |
| IM3 | A2, A11, B4001, B58, CW10, CW7 | Yes | No | ATI, SSCS, AVF, IED, SEN, VQP, TLD, VLK, GLC, YVL, SLR, CLG |
| IM4 | A11, A29, B7, B56, CW1, CW15 | Yes | No | ATI, SSCS, AVF, RPP, RPQG, IPQ |
| IM5 | A2, A11, B55, B62, CW1, CW0401/03/05/1801/02 | Yes | No | ATI, SSCS |
| IM6 | A2, A33, B38, B58, CW10, CW7 | Yes | No | TLD, VLK, GLC, YVL, SLR, CLG |
| IM7 | A2, A24, B4001, B35, CW4, CW15 | Yes | No | TLD, VLK, GLC, YVL, SLR, CLG, PYL, IACP, VMS, TYG, TYP, PYS, IED, SEN, HPV, EPL |
| IM8 | A11, A31, B4001, B40, CW3, CW7 | Yes | No | ATI, SSCS, AVF, IED, SEN |
| IM9 | A2, A11, B46, B51, CW1, CW15 | Yes | No | TLD, VLK, GLC, YVL, SLR, CLG, ATI, SSCS, AVF, VQP |
| IM10 | A11, B4001, B54, CW1, CW3 | Yes | No | ATI, SSCS, AVF, IED |
| IM11 | A11, B15, B58, CW0302/14, CW0801/03/06/08 | Yes | No | ATI, SSCS, AVF, VSF |
| IM12 | A3,A33, B13, B58, CW6, CW10 | Yes | No | VSF |
| IM13 | A2, A24, B48, B56, CW1, CW8 | Yes | No | VLK, GLC, YVL, SLR, CLG, PYL, IACP, VMS, TYG, TYP, RYS |
| IM14 | A2, A11, B4001, B13, CW7 | Yes | No | ATI, SSCS, AVF, IED, SEN, VQP, TLD, VLK, GLC, YVL, SLR, CLG |
| IM15 | A11, A33, B15, B58, CW9, CW10 | Yes | No | ATI, SSCS, VSF |
| IM16 | A2, A26, B46, CW1 | Yes | No | TLD, VLK, GLC, YVL, SLR, CLG, VQP |
| IM17 | A1, A11, B4001, B13, CW7, CW10 | Yes | No | ATI, SSCS,AVF, IED, SEN |

| | | | | |
|---|---|---|---|---|
| IM18 | A2, A11, B38, B75 | Yes | Yes | TLD, VLK, GLC, YVL, SLR, CLG, ATI, SSCS, AVF |
| IM19 | Not done | No | Yes | *Overlapping peptides |
| IM20 | Not done | No | Yes | *Overlapping peptides |
| IM21 | Not done | No | Yes | *Overlapping peptides |
| IM22 | Not done | No | Yes | *Overlapping peptides |
| IM23 | Not done | No | Yes | *Overlapping peptides |
| IM24 | Not done | No | Yes | *Overlapping peptides |
| IM25 | Not done | No | Yes | *Overlapping peptides |
| IM26 | Not done | No | Yes | *Overlapping peptides |
| IM27 | Not done | No | Yes | *Overlapping peptides |
| IM28 | Not done | No | Yes | *Overlapping peptides |
| IM29 | Not done | No | Yes | *Overlapping peptides |
| AS1 | A2, A11, B60, B46, CW7, CW1 | No | No | TLD, VLK, GLC, YVL, SLR, CLG, ATI, SSCS, AVF, IED, SEN, VQP |
| AS2 | A11, A33, B60, B44, C7, C3 | No | No | TLD, VLK, GLC, YVL, SLR, CLG |
| AS3 | A11, A33, B44, B75, C7, C8 | No | No | ATI, SSCS, AVF |
| AS4 | A2, A24, B55, B67, C7, C1 | No | No | TLD, VLK, GLC, YVL, SLR, CLG, PYL, IACP, VMS, TYG, TYP, RYS |
| AS5 | A11, B40, C3, C7 | No | No | ATI, SSCS, AVF, IED, SEN |
| AS6 | A11, A33, B38, B58, C7, C10 | Yes | No | ATI, SSCS, AVF, VSF |
| AS7 | A1101, A33, B2704, B5801 | Yes | No | ATI, SSCS, AVF, VSF |
| AS8 | A2, A11, B60, B46, C7, C1 | Yes | No | TLD, VLK, GLC, YVL, SLR, CLG, ATI, SSCS, AVF, IED, SEN, VQP |
| AS9 | A2, A26, B35, B51, C4, C14 | Yes | No | TLD, VLK, GLC, YVL, SLR, CLG |
| AS10 | A11, A33, B58, B60, C7, C10 | Yes | No | ATI, SSCS, AVF, IED, SEN, VSF |

| | | | | |
|---|---|---|---|---|
| AS11 | A11, B13, B62, C3, C4 | Yes | No | ATI, SSCS, AVF |
| AS12 | A11, A33, B27, B58, C10, C12 | Yes | No | ATI, SSCS, AVF, VSF |

*Four lytic 15-mer overlapping peptides including BMLF1, BRLF1, BZLF1, GP350 and five latent 15-mer overlapping peptides including EBNA1, EBNA3A, EBNA3B, EBNA3C and LMP2 were used in flow cytometric assays.
