# Supplementary material for: Emergence of CD4+ and CD8+ Polyfunctional T Cell Responses Against Immunodominant Lytic and Latent EBV Antigens in Children With Primary EBV Infection
Source: Front Microbiol. 2018 Mar 7;9:416. doi: 10.3389/fmicb.2018.00416 (PMC5863510; doi:10.3389/fmicb.2018.00416)
Supplement: Supplementary file 5 [file Image_2.PDF]

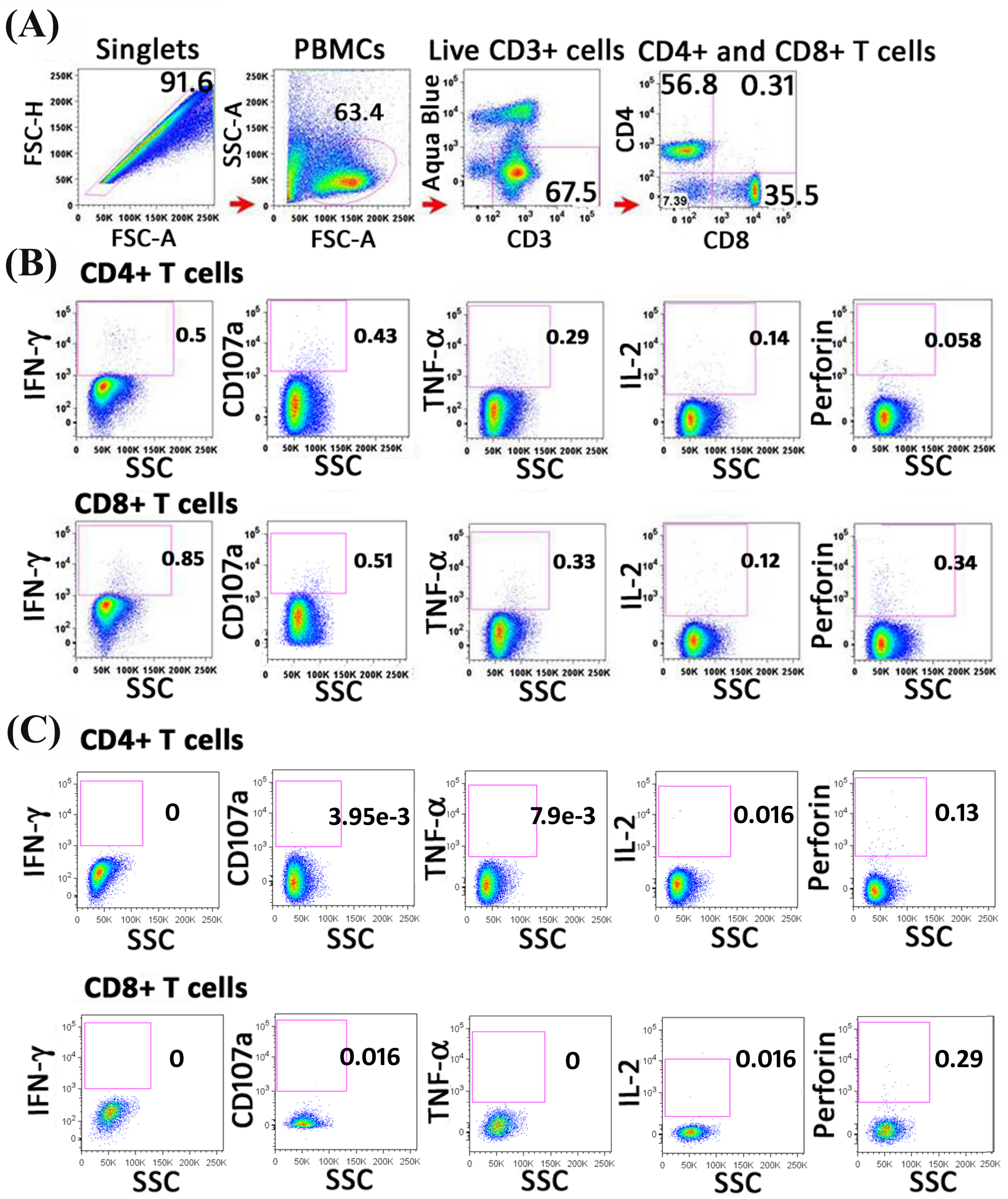

Supplementary Figure 2. Polychromatic flow cytometry for assessment of EBV-specific T cell responses in one representative IM patient and one EBV seronegative individual. **(A)** Gating strategy applied to identify the EBV-specific CD4+ and CD8+ T cells. **(B)** Analysis on multiple functions of CD4+ and CD8+ T cell responses towards the overlapping peptide pool of BZLF1 at the time of 1 month after diagnosis in one representative IM patient. **(C)** CD4+ and CD8+ T cell responses to EBNA3A in one EBV seronegative individual. No cytokine production was detected in the EBV seronegative individual after stimulation with EBV overlapping peptide pools.
