# Supplementary material for: Emergence of CD4+ and CD8+ Polyfunctional T Cell Responses Against Immunodominant Lytic and Latent EBV Antigens in Children With Primary EBV Infection
Source: Front Microbiol. 2018 Mar 7;9:416. doi: 10.3389/fmicb.2018.00416 (PMC5863510; doi:10.3389/fmicb.2018.00416)
Supplement: Supplementary file 6 [file Image_3.PDF]

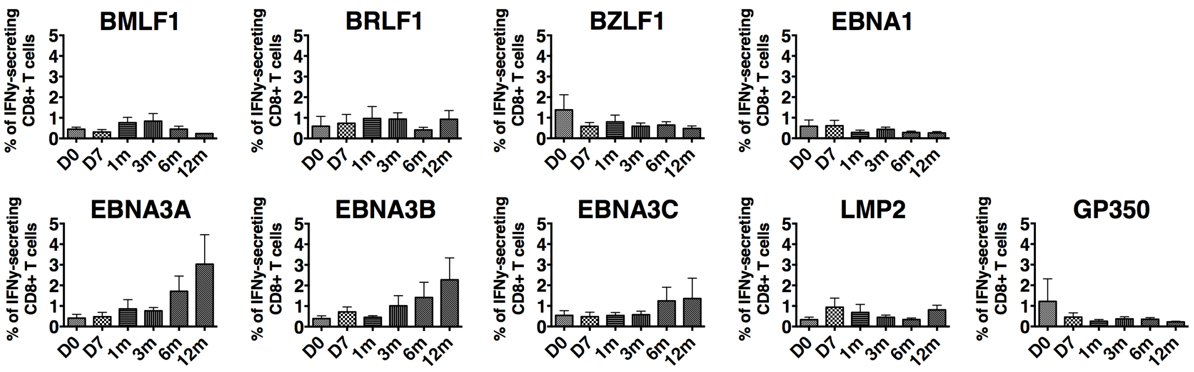

Supplementary Figure 3. Proportion of EBV-specific interferon-gamma producing CD8+ T cells towards EBV lytic and latent antigen peptides detected by flow cytometry. The mean +/- SEM of T cell responses in IM19-29 along 6 time points was shown. Decreases in T cell responses towards EBV lytic peptide pools (BZLF1, GP350) together with increases in T cell responses towards EBV latent peptide pools (EBNA3A, EBNA3B, EBNA3C) were identified in flow cytometric assays.
