# Supplementary material for: Emergence of CD4+ and CD8+ Polyfunctional T Cell Responses Against Immunodominant Lytic and Latent EBV Antigens in Children With Primary EBV Infection
Source: Front Microbiol. 2018 Mar 7;9:416. doi: 10.3389/fmicb.2018.00416 (PMC5863510; doi:10.3389/fmicb.2018.00416)
Supplement: Supplementary file 7 [file Image_4.PDF]

(A)

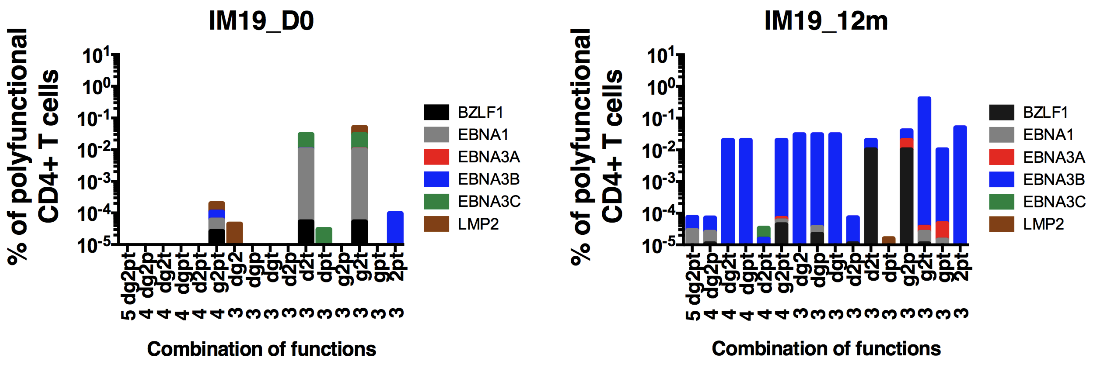

IM19_D0
IM19_12m
% of polyfunctional CD4+ T cells
Combination of functions
5 dg2pt
4 dg2p
4 dg2t
4 dgpt
4 d2pt
4 g2pt
3 dg2
3 dgp
3 dgt
3 d2p
3 d2t
3 dpt
3 g2p
3 g2t
3 gpt
3 2pt
BZLF1
EBNA1
EBNA3A
EBNA3B
EBNA3C
LMP2

(B)

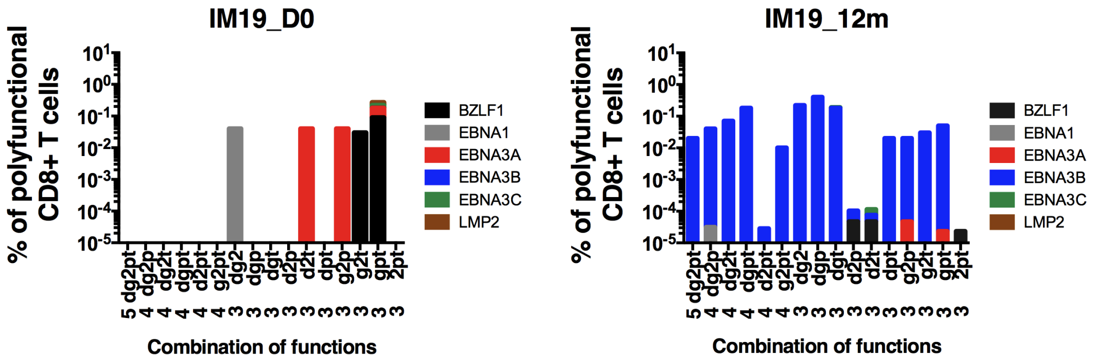

IM19_D0
IM19_12m
% of polyfunctional CD8+ T cells
Combination of functions
BZLF1
EBNA1
EBNA3A
EBNA3B
EBNA3C
LMP2

(C)

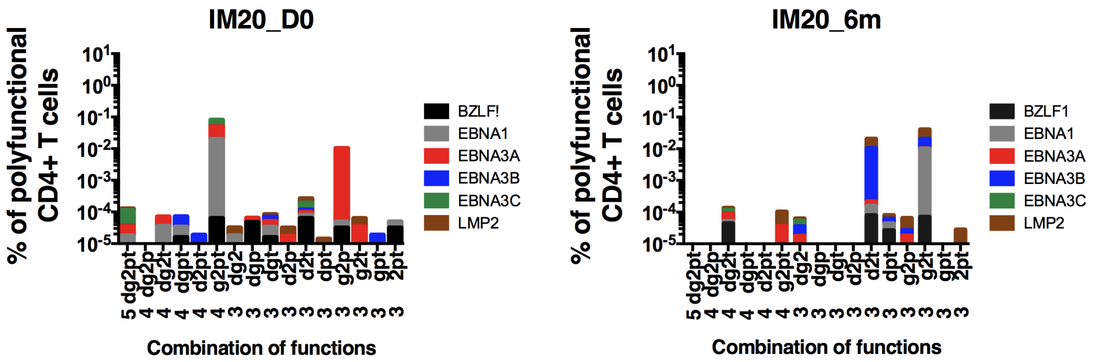

IM20_D0
IM20_6m
% of polyfunctional CD4+ T cells
Combination of functions
BZLF!
BZLF1
EBNA1
EBNA3A
EBNA3B
EBNA3C
LMP2

(D)

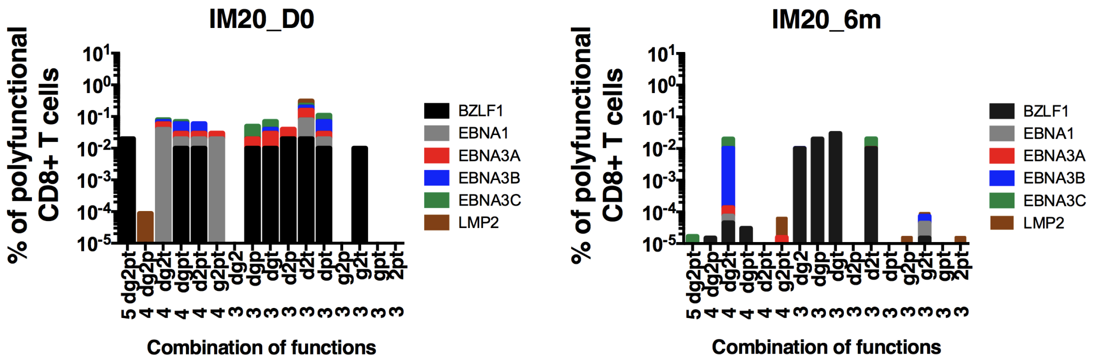

IM20_D0
IM20_6m
% of polyfunctional CD8+ T cells
Combination of functions
BZLF1
EBNA1
EBNA3A
EBNA3B
EBNA3C
LMP2

(E)

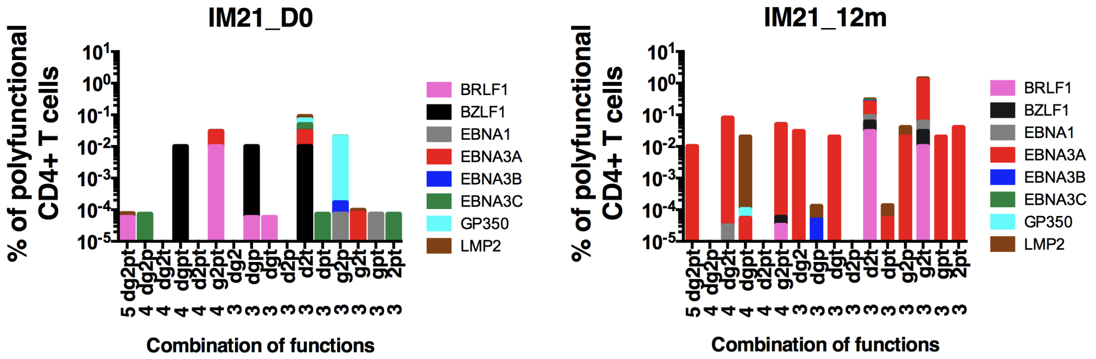
IM21_D0
IM21_12m
% of polyfunctional CD4+ T cells
10^{1} 10^{0} 10^{-1} 10^{-2} 10^{-3} 10^{-4} 10^{-5}
5 dg2pt
4 dg2p
4 dg2t
4 dgpt
4 d2pt
4 g2pt
3 dg2
3 dgp
3 dgt
3 d2p
3 d2t
3 dpt
3 g2p
3 g2t
3 gpt
3 2pt
Combination of functions
BRLF1
BZLF1
EBNA1
EBNA3A
EBNA3B
EBNA3C
GP350
LMP2

(F)

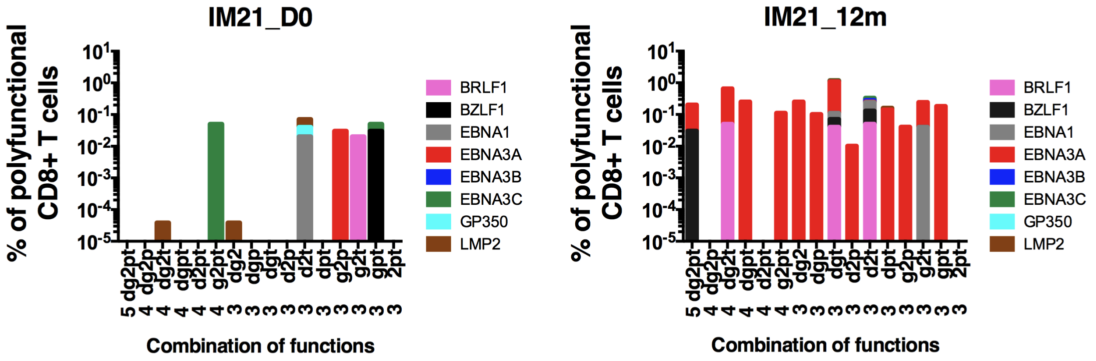
IM21_D0
IM21_12m
% of polyfunctional CD8+ T cells
10^{1} 10^{0} 10^{-1} 10^{-2} 10^{-3} 10^{-4} 10^{-5}
5 dg2pt
4 dg2p
4 dg2t
4 dgpt
4 d2pt
4 g2pt
3 dg2
3 dgp
3 dgt
3 d2p
3 d2t
3 dpt
3 g2p
3 g2t
3 gpt
3 2pt
Combination of functions
BRLF1
BZLF1
EBNA1
EBNA3A
EBNA3B
EBNA3C
GP350
LMP2

(G)

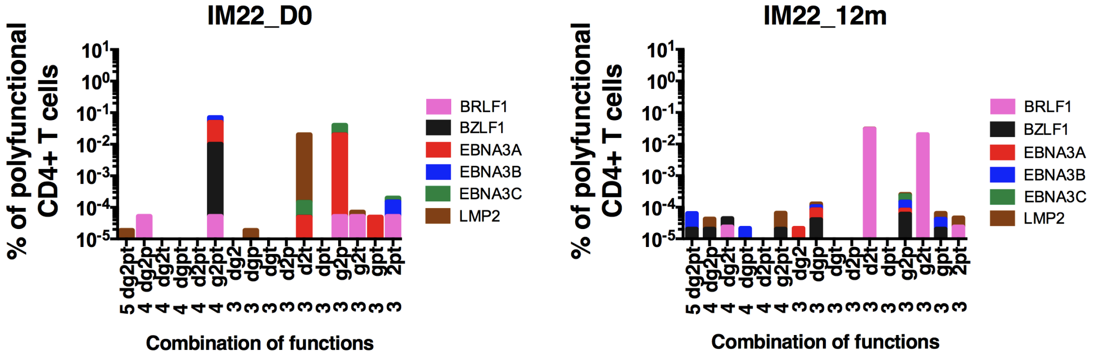
IM22_D0
IM22_12m
% of polyfunctional CD4+ T cells
10^{1} 10^{0} 10^{-1} 10^{-2} 10^{-3} 10^{-4} 10^{-5}
5 dg2pt
4 dg2p
4 dg2t
4 dgpt
4 d2pt
4 g2pt
3 dg2
3 dgp
3 dgt
3 d2p
3 d2t
3 dpt
3 g2p
3 g2t
3 gpt
3 2pt
Combination of functions
BRLF1
BZLF1
EBNA3A
EBNA3B
EBNA3C
LMP2

(H)

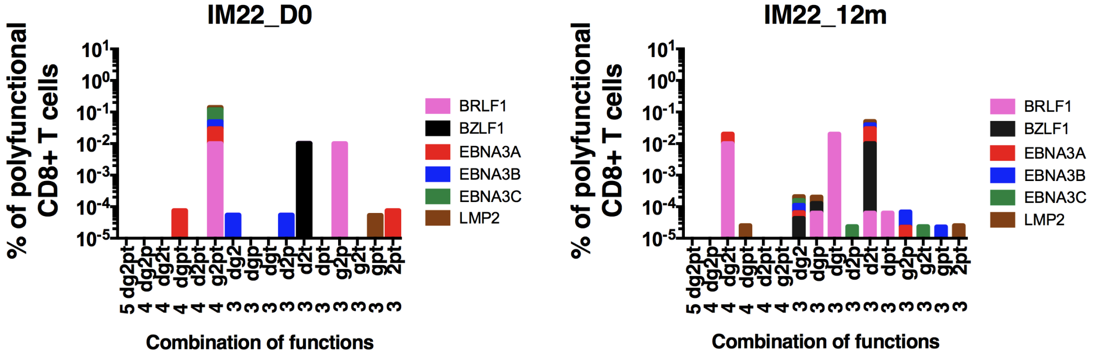
IM22_D0
IM22_12m
% of polyfunctional CD8+ T cells
10^{1} 10^{0} 10^{-1} 10^{-2} 10^{-3} 10^{-4} 10^{-5}
5 dg2pt
4 dg2p
4 dg2t
4 dgpt
4 d2pt
4 g2pt
3 dg2
3 dgp
3 dgt
3 d2p
3 d2t
3 dpt
3 g2p
3 g2t
3 gpt
3 2pt
Combination of functions
BRLF1
BZLF1
EBNA3A
EBNA3B
EBNA3C
LMP2

(I)

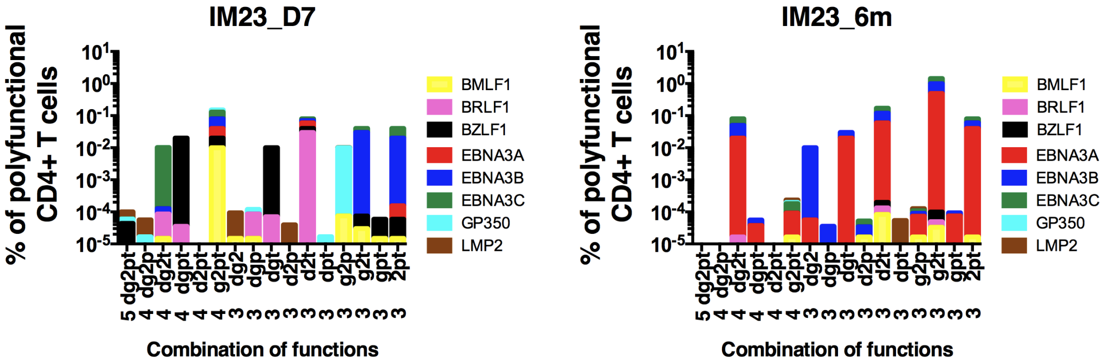
IM23_D7
IM23_6m
% of polyfunctional CD4+ T cells
BMLF1
BRLF1
BZLF1
EBNA3A
EBNA3B
EBNA3C
GP350
LMP2
5 dg2pt
4 dg2p
4 dg2t
4 dgpt
4 d2pt
4 g2pt
3 dg2
3 dgp
3 dgt
3 d2p
3 d2t
3 dpt
3 g2p
3 g2t
3 gpt
3 2pt
Combination of functions

(J)

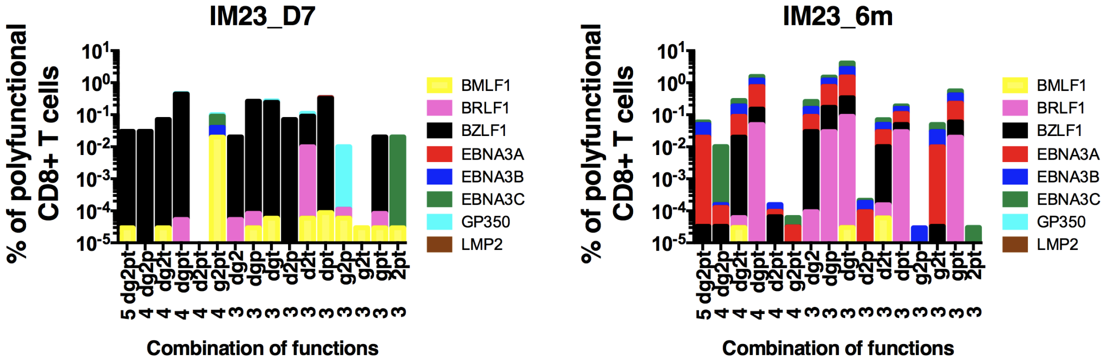
IM23_D7
IM23_6m
% of polyfunctional CD8+ T cells
BMLF1
BRLF1
BZLF1
EBNA3A
EBNA3B
EBNA3C
GP350
LMP2
Combination of functions

(K)

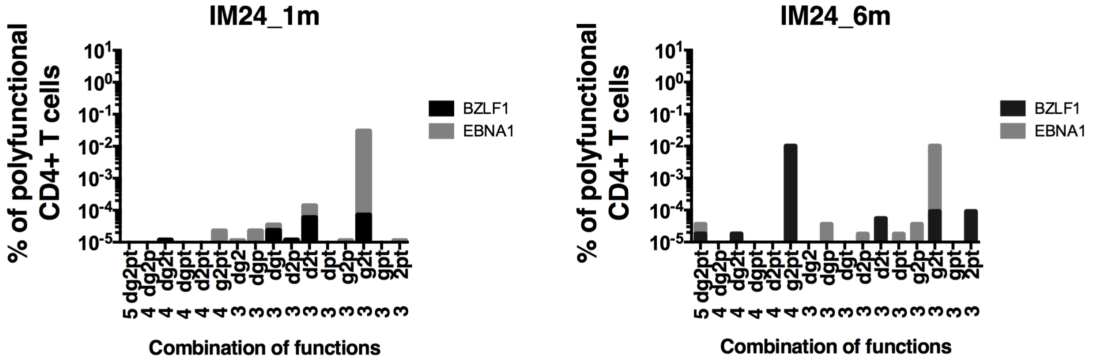
IM24_1m
IM24_6m
% of polyfunctional CD4+ T cells
BZLF1
EBNA1
Combination of functions

(L)

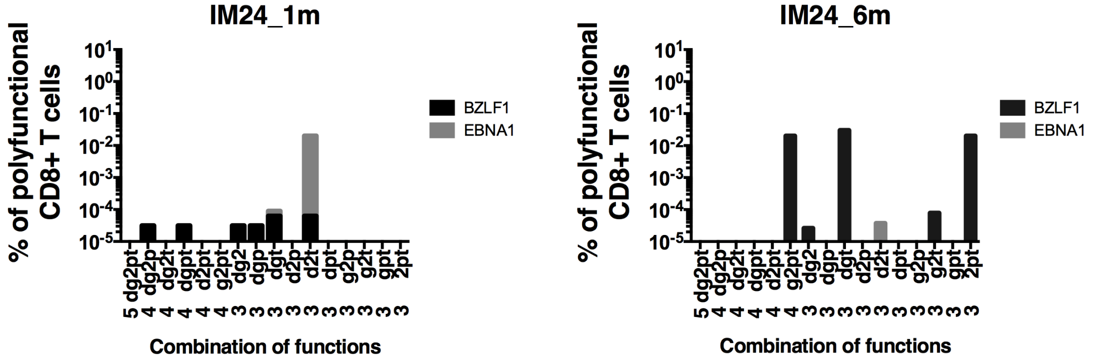
IM24_1m
IM24_6m
% of polyfunctional CD8+ T cells
BZLF1
EBNA1
Combination of functions

(M)

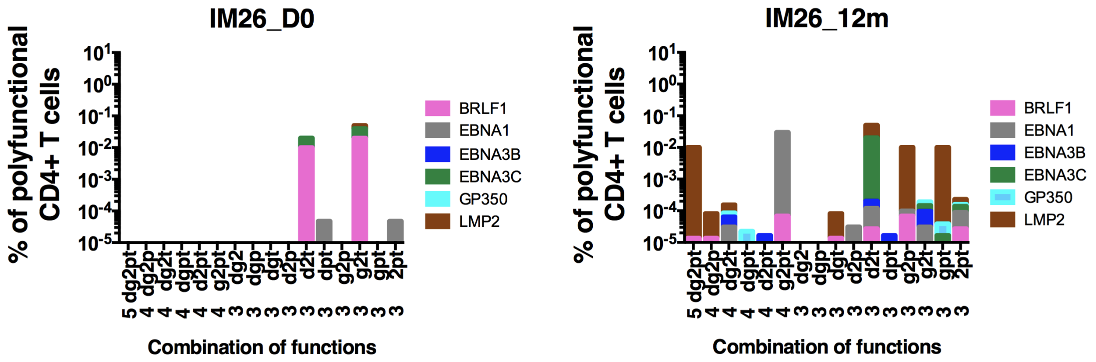
IM26_D0
IM26_12m
% of polyfunctional CD4+ T cells
Combination of functions
BRLF1
EBNA1
EBNA3B
EBNA3C
GP350
LMP2

(N)

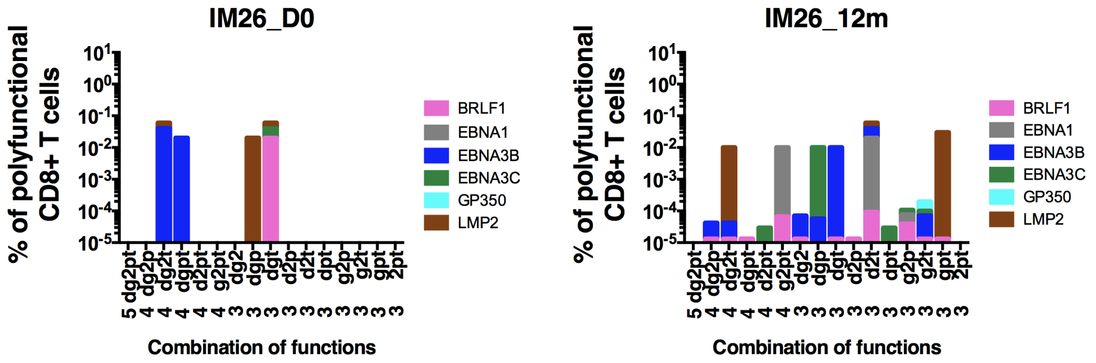
IM26_D0
IM26_12m
% of polyfunctional CD8+ T cells
Combination of functions
BRLF1
EBNA1
EBNA3B
EBNA3C
GP350
LMP2

(O)

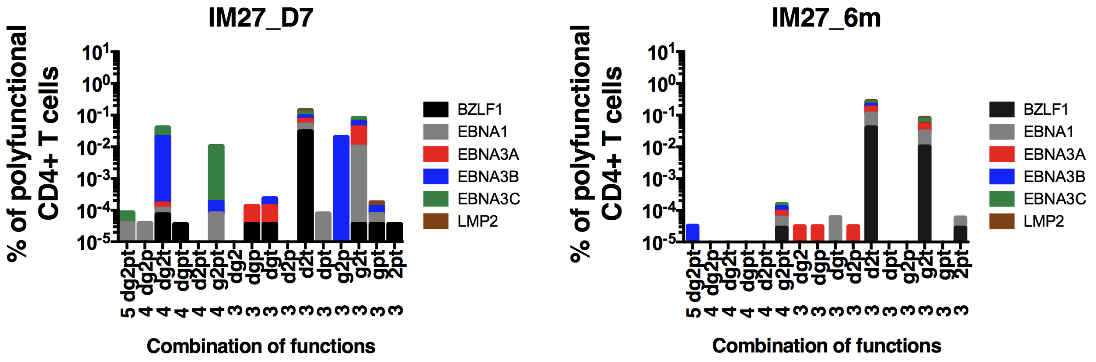
IM27_D7
IM27_6m
% of polyfunctional CD4+ T cells
Combination of functions
BZLF1
EBNA1
EBNA3A
EBNA3B
EBNA3C
LMP2

(P)

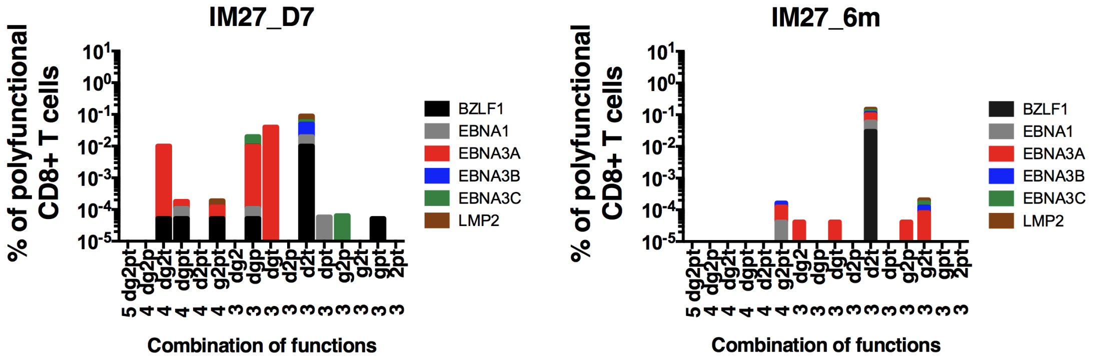
IM27_D7
IM27_6m
% of polyfunctional CD8+ T cells
Combination of functions
BZLF1
EBNA1
EBNA3A
EBNA3B
EBNA3C
LMP2

(Q)

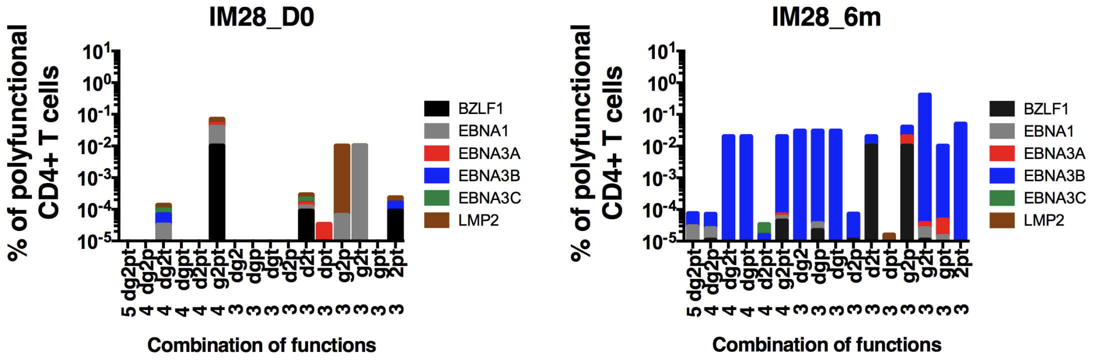
IM28_D0
IM28_6m
% of polyfunctional CD4+ T cells
10^{1} 10^{0} 10^{-1} 10^{-2} 10^{-3} 10^{-4} 10^{-5}
5 dg2pt 4 dg2p 4 dg2t 4 dgpt 4 d2pt 4 g2pt 3 dg2 3 dgp 3 dgt 3 d2p 3 d2t 3 dpt 3 g2p 3 g2t 3 gpt 3 2pt
Combination of functions
BZLF1
EBNA1
EBNA3A
EBNA3B
EBNA3C
LMP2

(R)

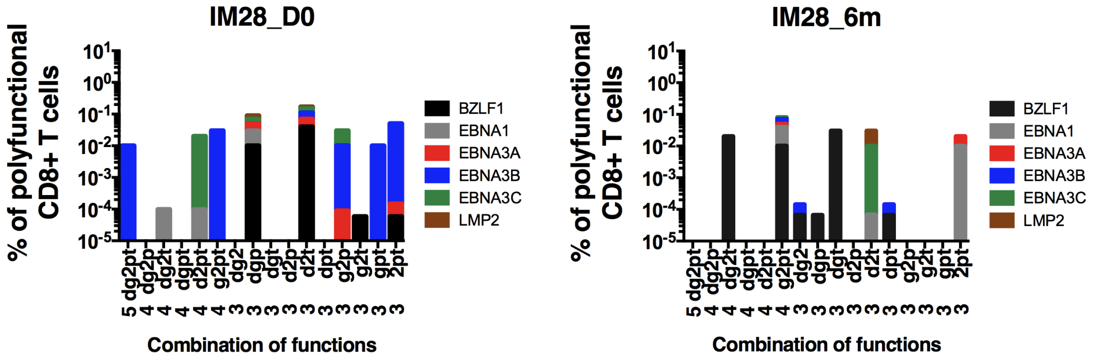
IM28_D0
IM28_6m
% of polyfunctional CD8+ T cells
10^{1} 10^{0} 10^{-1} 10^{-2} 10^{-3} 10^{-4} 10^{-5}
5 dg2pt 4 dg2p 4 dg2t 4 dgpt 4 d2pt 4 g2pt 3 dg2 3 dgp 3 dgt 3 d2p 3 d2t 3 dpt 3 g2p 3 g2t 3 gpt 3 2pt
Combination of functions
BZLF1
EBNA1
EBNA3A
EBNA3B
EBNA3C
LMP2

(S)

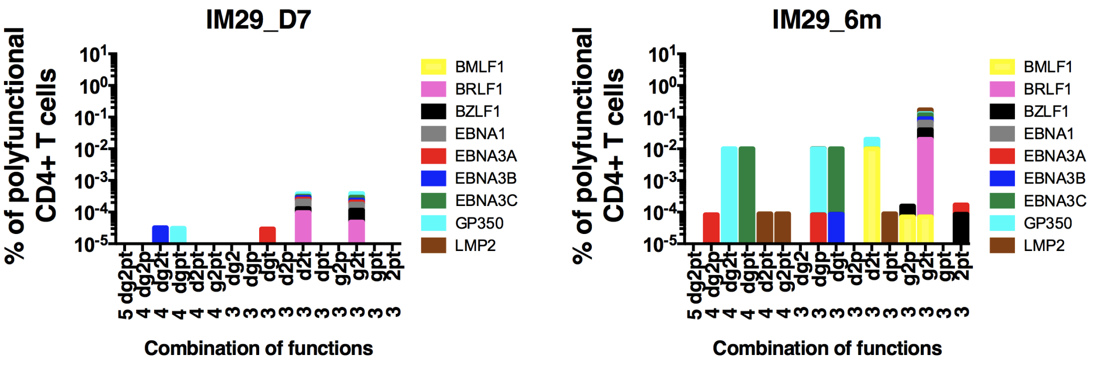
IM29_D7
IM29_6m
% of polyfunctional CD4+ T cells
10^{1} 10^{0} 10^{-1} 10^{-2} 10^{-3} 10^{-4} 10^{-5}
5 dg2pt 4 dg2p 4 dg2t 4 dgpt 4 d2pt 4 g2pt 3 dg2 3 dgp 3 dgt 3 d2p 3 d2t 3 dpt 3 g2p 3 g2t 3 gpt 3 2pt
Combination of functions
BMLF1
BRLF1
BZLF1
EBNA1
EBNA3A
EBNA3B
EBNA3C
GP350
LMP2

(T)

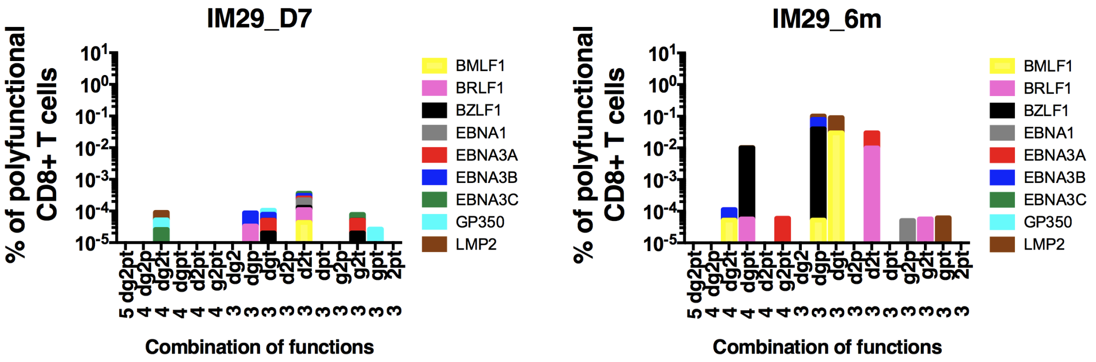
IM29_D7
IM29_6m
% of polyfunctional CD8+ T cells
10^{1} 10^{0} 10^{-1} 10^{-2} 10^{-3} 10^{-4} 10^{-5}
5 dg2pt 4 dg2p 4 dg2t 4 dgpt 4 d2pt 4 g2pt 3 dg2 3 dgp 3 dgt 3 d2p 3 d2t 3 dpt 3 g2p 3 g2t 3 gpt 3 2pt
Combination of functions
BMLF1
BRLF1
BZLF1
EBNA1
EBNA3A
EBNA3B
EBNA3C
GP350
LMP2

Supplementary Figure 4. Evolution of the functionality of CD4+ and CD8+ T cells towards the EBV lytic and latent overlapping peptide pools in 11 IM patients from acute infection to a long time point (either 6 or 12 months post-diagnosis). The percentages of responsive **(A & B) CD4+ and CD8+ T cells in IM19 (C & D) CD4+ and CD8+ T cells in IM20 (E & F) CD4+ and CD8+ T cells in IM21 (G & H) CD4+ and CD8+ T cells in IM22 (I & J) CD4+ and CD8+ T cells in IM23 (K & L) CD4+ and CD8+ T cells in IM24 (M & N) CD4+ and CD8+ T cells in IM26 (O & P) CD4+ and CD8+ T cells in IM27 (Q & R) CD4+ and CD8+ T cells in IM28 (S & T) CD4+ and CD8+ T cells in IM29** with different combinations of functions upon stimulation by BMLF1, BRLF1, BZLF1, EBNA1, EBNA3A, EBNA3B and EBNA3C at acute infection (from diagnosis to 1 month post diagnosis, D0 to 1m) to a long time point (either 6 or 12 months after diagnosis, 6 to 12m). d, CD107a; g, Interferon-gamma; 2, Interleukin-2; p, Perforin; t, Tumor-necrosis factor-alpha.
