# Supplementary material for: Emergence of CD4+ and CD8+ Polyfunctional T Cell Responses Against Immunodominant Lytic and Latent EBV Antigens in Children With Primary EBV Infection
Source: Front Microbiol. 2018 Mar 7;9:416. doi: 10.3389/fmicb.2018.00416 (PMC5863510; doi:10.3389/fmicb.2018.00416)
Supplement: Supplementary file 4 [file Image_1.PDF]

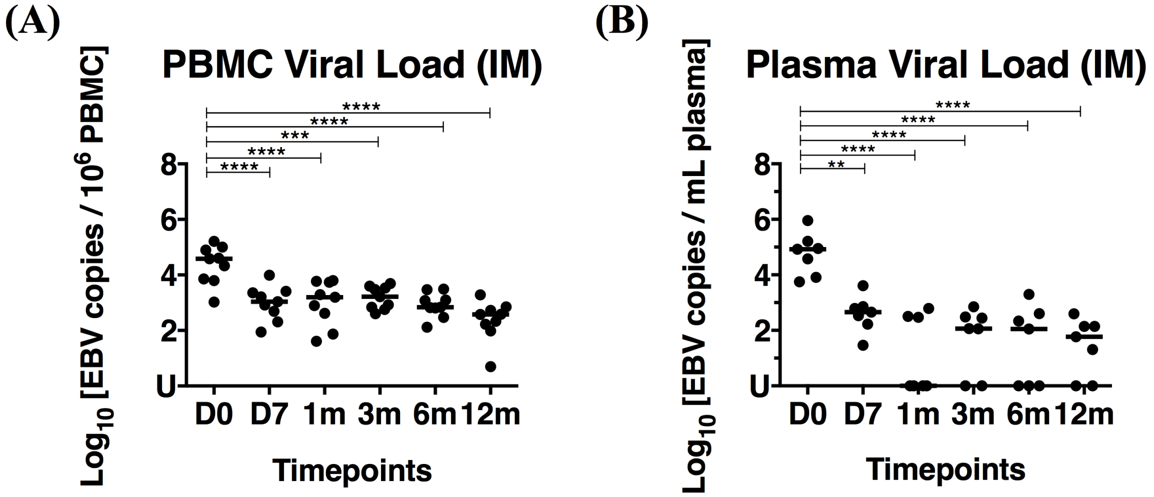

Supplementary Figure 1. Statistical analyses of PBMC and plasma viral loads in IM patients. (A) EBV loads in PBMC of 9 IM patients. **(B)** EBV loads in plasma of 7 IM patients. The spots represent the copy numbers of EBV per million PBMC and copy numbers of EBV per mL plasma in different IM patients from the day of diagnosis (D0) to 12 months after diagnosis (12m). Only cases with completed set of time points (D0, D7, 1m, 3m, 6m and 12m) in PBMC or plasma viral load were analyzed by repeated measure ANOVA and Bonferroni's correction for multiple comparisons. The viral loads at D7, 1m, 3m, 6m and 12m were significantly lower than D0. "**", "***" and "****" represent P-value < 0.01, < 0.001 and < 0.0001, respectively. U, undetectable.
